# Physiological and transcriptomic analyses characterized high temperature stress response mechanisms in *Sorbus pohuashanensis*

**DOI:** 10.1038/s41598-021-89418-7

**Published:** 2021-05-12

**Authors:** Xin Pei, Yan Zhang, Lingyi Zhu, Dongxue Zhao, Yizeng Lu, Jian Zheng

**Affiliations:** 1 School of Landscape Architecture, Beijing University of Agriculture, Beijing, 102206 China; 2 Shandong Provincial Center of Forest Tree Germplasm Resources, Shandong Province, Jinan, 250102 China; 3 Beijing Laboratory of Urban and Rural Ecological Environment, Beijing, 100083 China

**Keywords:** Plant domestication, Plant sciences, Plant stress responses, Heat

## Abstract

*Sorbus pohuashanensis* (Hance) Hedl. is a Chinese native alpine tree species, but the problem of introducing *S. pohuashanensis* to low altitude areas has not been solved. In this study, we aimed to explore the molecular regulatory network of *S*. *pohuashanensis* in response to high-temperature stress using RNA-Sequencing technology and physiological and biochemical determination. Based on transcriptomic data, we obtained 1221 genes (752 up-regulated and 469 down-regulated) that were differentially expressed during 8 h 43℃ treatment and candidate genes were related to calcium signaling pathway, plant hormone signal transduction, heat shock factors, chaperones, ubiquitin mediated proteolysis, cell wall modification, ROS scavenging enzymes, detoxification and energy metabolism. The analysis of high temperature response at the physiological level and biochemical level were performed. The chlorophyll fluorescence parameters of leaf cells decreased, the content of osmotic regulators increased, and the activity of ROS scavenging enzymes decreased. The molecular regulatory network of *S*. *pohuashanensis* in response to high-temperature stress was preliminarily revealed in this study, which provides fundamental information improving introducing methods and discovering heat-tolerant genes involved in high-temperature stress in this species and provides a reference for other plants of the genus *Sorbus*.

## Introduction

Introduction and acclimatization is the nearest way to enrich species and breeding materials, which is widely used worldwide^1^. The introduction and acclimatization of alpine plants has always been one of the difficulties in forest genetics^2^. Wild species of alpine plants mainly grow in the mountains at an altitude of several thousand meters^3^. Due to the mountain forest environment, alpine plants have special requirements for light, temperature, water, soil and other conditions, and temperature, especially high-temperature, is one of the most important factors that restrict the success of introduction and acclimatization^4^. Heat stress can be classified into moderate and extreme according to duration of stress, time of the day at which it occurs and co-exposure to other stresses, each of which involves different coping mechanisms and adaptation strategies^5^. Such temperature stress occurs regularly in alpine environments^6^. Recently, increasing numbers of reports have indicated that with climate warming, the high temperature heat wave will last longer and will occur more frequently^7^. In addition, plants are more often exposed to severe high-temperature stress because they are fixed and high-temperature affects the physiological and ecological processes of plants^8^. Therefore, studying and discussing the thermal tolerance of plants and discussing how to improve the thermal tolerance of plants has become one of the important topics to cope with global warming, and has gradually received widespread attention. Previous reports have shown that attempts to introduce alpine plants have made some advance. For example, the tolerance of *Trollius chinensis* to extreme high temperatures in summer can be improved by shading seedlings after being introduced to the Beijing plain from the alpine mountainous area of 1500–2600 m above sea level^9^. The high temperature and drought in the introduced area greatly affect the growth and development of *Rhododendron lapponicum*. However, the stress resistance of *R*. *lapponicum* can be increased by breeding varieties with better heat tolerance^10^. But the specific mechanisms that influence the heat resistance of alpine plants after introduction to the plains are still unclear.

Existing studies show that adverse effects of high-temperature stress on plants mainly include inhibition of seed germination, reduction of plant growth and reproduction, and reduction of crop yield and quality^11^. High temperature stress affects all aspects of plant life, with photosynthesis taking the brunt of the stress and being highly susceptible to heat-mediated damage.Though several chloroplast proteins get denatured or become nonfunctional at high-temperature, the photosystem-II (PSII) is most vulnerable to get damaged^12^. High-temperature damages the chloroplast and mitochondrial electron transport system. It generates ROS such as superoxides, hydrogen peroxide (H_2_O_2_), and hydroxyl radicals (·OH) that harm DNA and also cause lipid peroxidation of the cell membrane^13^. Plant response to high-temperature stress is relatively conservative^14^. Previous studies have revealed some key factors in the plant response system to high-temperature stress, which together constitute the main regulatory network of plant response to high-temperature stress. Plant response to high-temperature stress regulation network including small G protein/G protein mediated calcium signaling pathways, hormone regulation networks, heat shock transcription factors (Hsfs) and heat shock proteins (Hsps) reaction and reactive oxygen species (ROS) reaction, and these factors interact together to complete the response of high-temperature stress^15^. Plant cells first perceive high-temperature stimulation and amplify intracellular signals, then induce the expression of heat-resistant genes. Signal transduction and transcription regulation networks are crucial in the process of plant responses to high-temperature stress^16^. Calcium and calmodulin (CaM) are locatedupstream of the high temperature signal transmission chain and have the characteristics of rapidity and broad spectrum. High-temperature triggered signals promote calcium to bind to CaM, further activate calcium-dependent protein kinase (CDPK), and induce mitogen-activated protein kinases (MAPKs) transmit signals to the nucleus and ultimately induce heat-tolerant gene expression^17^. Heat shock transcription factor A1s (HsfA1s) is considered to be a key "regulator" of thermotolerance, which can activate the expression of heat shock response (HSR) genes, and then regulate the synthesis of molecular chaperones and enzymes involved in unfolded protein degradation and ROS scavenging^18^. Hsps play the most crucial role in the several intricate mechanisms which plants protect themselves from excessive high-temperature stress^19,20^. High temperatures lead to the accumulation of unfolded proteins in plant cells, which must be regenerated or degraded by plants in order to maintain normal life activities^21^. By functioning as molecular chaperones, Hsps prevent protein denaturation and aggregation. In addition, Hsps interact and modulates functions of heat shock factors (HSFs) e.g. HsfAs, DREB, bZIP and WRKY^22^. ROS primarily functions as signal transduction molecules that regulate different pathways during plant acclimation to stress, but are also toxic byproducts of stress metabolism^13^. Accumulation of ROS in plants activates HSFs, which in turn activate ROS scavenging and detoxifying enzymes like ascorbate peroxidase (APX) and superoxide dismutase (SOD)^13^. Catalase (CAT) are also considered to be ROS scavenging enzymes necessary for ROS detoxification^23^. A decrease in ROS level occurs due to the enhanced production of antioxidants, osmolytes, and Hsps. The major stress-responsive osmolytes in plants include proline (Pro), glycine betaine, and trehalose, which play roles in maintaining cellular ionic homeostasis^24^. In addition, plant tissues also generate adaptive structures to resist the adverse effects of high-temperature. As a powerful dynamic barrier, cell wall can make metabolic and structural adaptations to the physiological changes of cells caused by high temperature stress, including the formation of cell wall modifications such as the cuticle in the aerial part and suberin in the root, and produce a special phenol ester-based protection system^25^.

*Sorbus pohuashanensis* (Hance) Hedl. , a small deciduous tree of the subfamily Maloideae of Rosaceae, is a native tree species and widely distributed at altitudes of 900–2,500 m in northern China^26^. With beautiful flowers, colorful fruits, and leaves of different colors in the four seasons, *S. pohuashanensis* has a very high ornamental significance in landscape^27^. In addition, *S. pohuashanensis* has specific medicinal and commercial significance^26,28^. Research on *S. pohuashanensis* has in recent years focused on plant secondary structure and affinities^29^, phylogenetic relationships of pink-fruit^30^, seed germination and dormancy characteristics^31^, nutritional and medicinal components of the fruit^26,32^, identification of pathogenic pathogens and development and application of SSR markers^27,33^. However, the main problem facing the development and utilization of *S. pohuashanensis* is the poor adaptability of introduction and acclimatization to the high temperature environment in summer in low altitude areas. Peng et al. analyzed the heat resistance physiological characteristics of *S*. *pohuashanensis* and found that it was sensitive to high-temperature stress^34^. At the molecular level, the mechanisms of high temperature response in *S. pohuashanensis* have been initially explored. Liu et al. sequenced the transcriptome of *S. pohuashanensis* and obtained a large number of Unigenes, from which *SpHsp70-1* was cloned to investigate the mechanisms and expression patterns in response to high temperature^27,35^. Zhang et al. cloned the small heat stress proteins(sHsps) *SpHsp17.3* and *SpHsp23.8* from *S. pohuashanensis* and investigated their tissue-specific expression and expression in response to abiotic stresses^36,37^. However, the molecular regulatory mechanisms of the response of *S*. *pohuashanensis* to high-temperature stress have not been elucidated. In this study, the differentially expressed genes (DEGs) of *S*. *pohuashanensis* were analyzed and the molecular regulatory networks were constructed during high-temperature treatment using RNA-seq technology to reveal the molecular response mechanisms to high temperature with determination of chloroplast fluorescence parameters, osmotic regulation substance content, and ROS scavenging enzyme activity. The results will contribute to the development and utilization of germplasm resources for *S*. *pohuashanensis* and provide an informatics basis for the genetic improvement and breeding of varieties of this species in the future.

## Results

### Phenotypes of *S. pohuashanensis* leaves under normal and high-temperature stress

**Figure 1:**
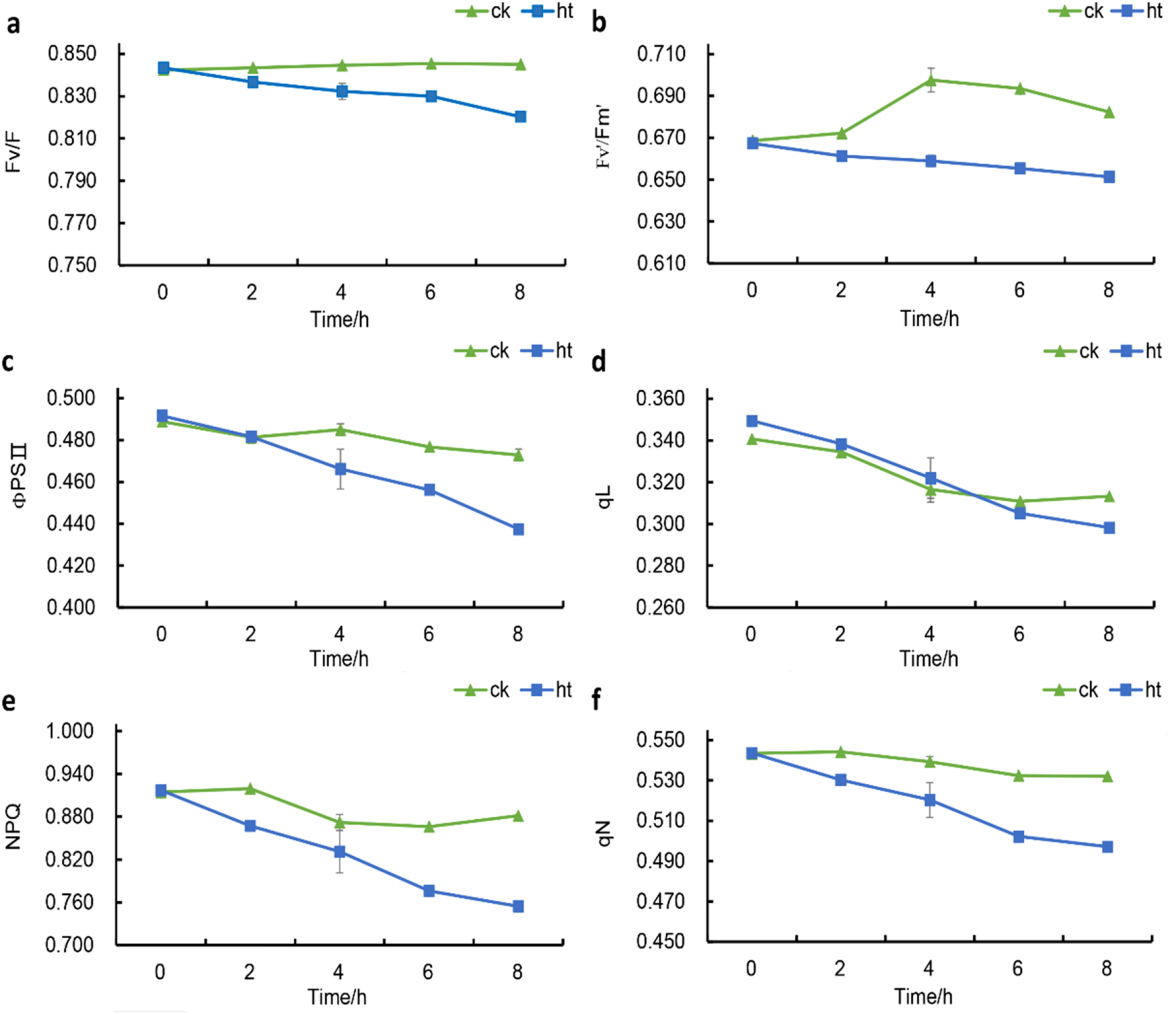
Effects of heat stress on the (**a**) Fv/Fm, (**b**) Fv`/Fm`, (**c**) ΦPSII, (**d**) qL, (**e**) NPQ and (**f**) qN of PS IIphotochemistry (significant difference at 5% levels according to standard deviation and multiple comparison).

The PSII process of dark reaction of photosynthesis in leaves of *S. pohuashanensis* in HT was affected to some extent within 0–8 h under 43℃ (Fig. 1). F_v_/F_m_ of leaves in HT was lower than that in CK from 0 h onwards, and F_v_/F_m_ of leaves in CK remained above 0.84 (Fig. 1a). The F_v_ '/F_m_'of the leaves in the HT showed a decreasing trend and was significantly lower than that in the CK, while the F_v_ '/F_m_' in the CK showed a rising and then decreasing trend (Fig. 1b). In addition, PSII of *S. pohuashanensis* leaves in HT showed a downward trend with the extension of time under high temperature stress, which was significantly lower than CK after 4 h of high temperature stress (Fig. 1c). There was no difference in qL between HT and CK in the first 6 h of high temperature stress, and HT was significantly lower than CK at 6–8 h of high temperature stress (Fig. 1d). It is worth mentioning that the NPQ and qN of the leaves in HT were always lower than those in CK after the beginning of high temperature stress, and gradually decreased with the extension of time; while the NPQ and qN in CK changed little within 0–8 h, and basically remained above 0.860 and 0.520 (Fig. 1e, f). The above results indicated that PSII process of HT was inhibited compared with CK.

**Figure 2:**
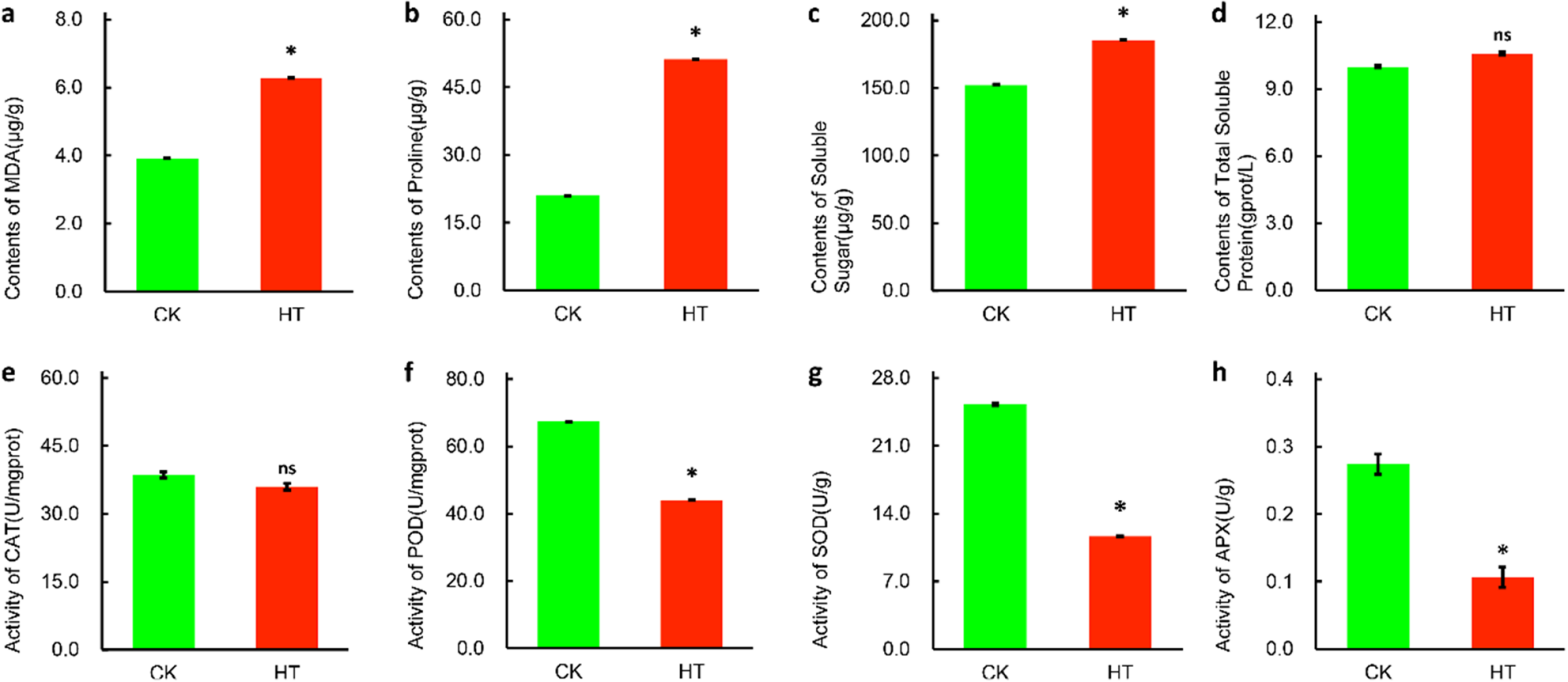
Physiological and biochemical indexes of leaves of *S. pahuashanensis* under heat stress. (**a**) MDA, (**b**) Pro, (**c**) Soluble sugar, (**d**) Total soluble protein, (**e**) CAT, (**f**) POD, (**g**) SOD and (**h**) APX (* indicate the significant difference at 5% levels according to student’s t-test, respectively. NS means no significant difference).

High temperature stress affected the content of osmotic regulatory substances in the leaves of *S. pohuashanensis*. After 8 h of high temperature stress, the content of malondialdehyde (MDA) in HT increased sharply, by 60.13% compared with CK (Fig. 2a) indicating the membrane lipid showed much higher degree of damage in HT. The Pro content in the leaves of *S. pohuashanensis* in HT also increased sharply, which was significantly different from the CK and 143.90% higher than that in CK (Fig. 2b), and the soluble sugar content and soluble protein content in HT were 17.79% and 5.75% higher than CK, respectively (Fig. 2c, d). Compared with CK, the content of osmoregulation substances in HT leaves increased.

High temperature stress also significantly affected the activity of ROS scavenging enzymes. Except for CAT, POD, SOD and APX activities of the leaf cells of *S. pohuashanensis* were significantly lower in HT than in CK after 8 h of high temperature stress (Fig. 2e, f, g, h).

### Transcriptome sequencing and de novo assembly

To elucidate the molecular responses to heat stress in *S. pohuashanensis*, six libraries were constructed from HT and CK samples for sequencing using the Illumina HiSeq2000 platform. In this study, approximately 53,461,517 raw reads and 51,558,437 clean reads were generated per sample (Supplementary Table S1). A total of 229,107 contigs were obtained after preliminary assembly. These contigs were assembled using the scaffolding algorithm (Word size = 45, Minimum contig length ≥ 200) of CLC Genomics Workbench software, and 197,028 primary unigenes were obtained. Finally, using CAP3 online stitching software, the primary unigenes were sequenced a second time and a transcriptome database for *S. pohuashanensis* was obtained. The de novo assembly generated 130,003 final unigenes, with an average length of 658 bp and an N50 of 838 bp (Supplementary Table S2). The completeness of the transcriptome assembly was assessed using BUSCO, which is based on evolutionarily informed expectations of gene content from eukaryota_odb10. Compared to the 255 single-copy orthologs for the embryophyta lineage, our assembly was 49.1% complete (107 complete single-copy and 18 complete duplicated BUSCO), while 37.3% of contigs were fragmented (95 BUSCOs) and 13.6% were missing (35 BUSCOs). These results indicated that the transcriptome assembly was useful for further transcriptomic analyses of *S. pohuashanensis* (Supplementary Fig. S1).

### GO and KEGG pathway analysis of DEGs

**Figure 3:**
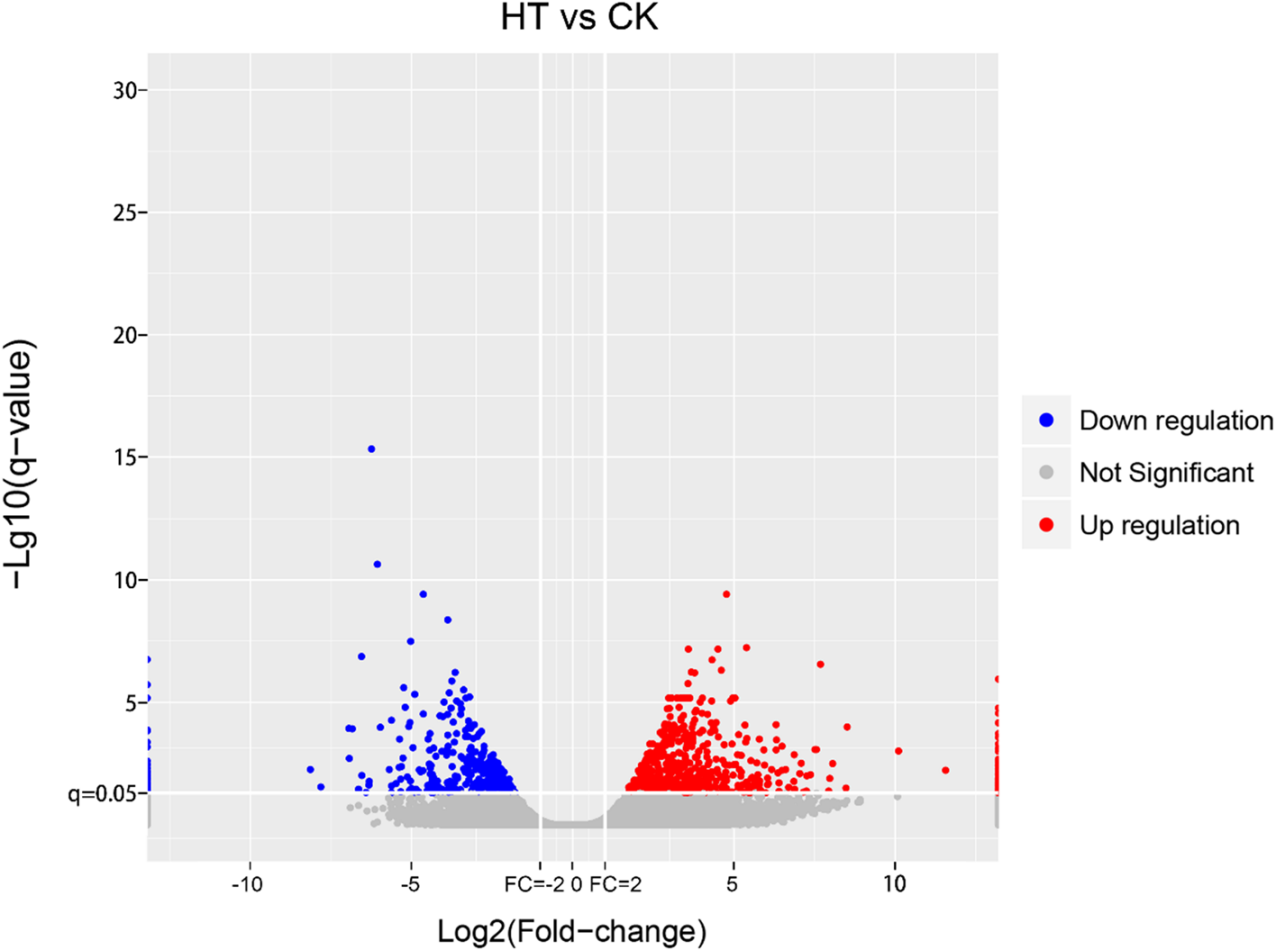
Volcano plot of differentially expressed transcripts with high-temperature stress treatment in *S. pohuashanensis* at q-value ≤ 0.05. Up-regulated and down-regulated genes were represented by red and blue dots, respectively.

FPKM was used to calculate the gene expression levels among different samples and to compare the mRNA levels of *S. pohuashanensis* response to high-temperature stress (Supplementary Table S1). DEGs (fold change ≥ 2 and q-value ≤ 0.05) were defined as genes that were abundant or rare in one sample relative to the other sample. The results showed that 1221 DEGs were identified in *S. pohuashanensis* after high-temperature stress (752 upregulated and 469 downregulated) (Fig. 3; Supplementary Table S4).

**Figure 4:**
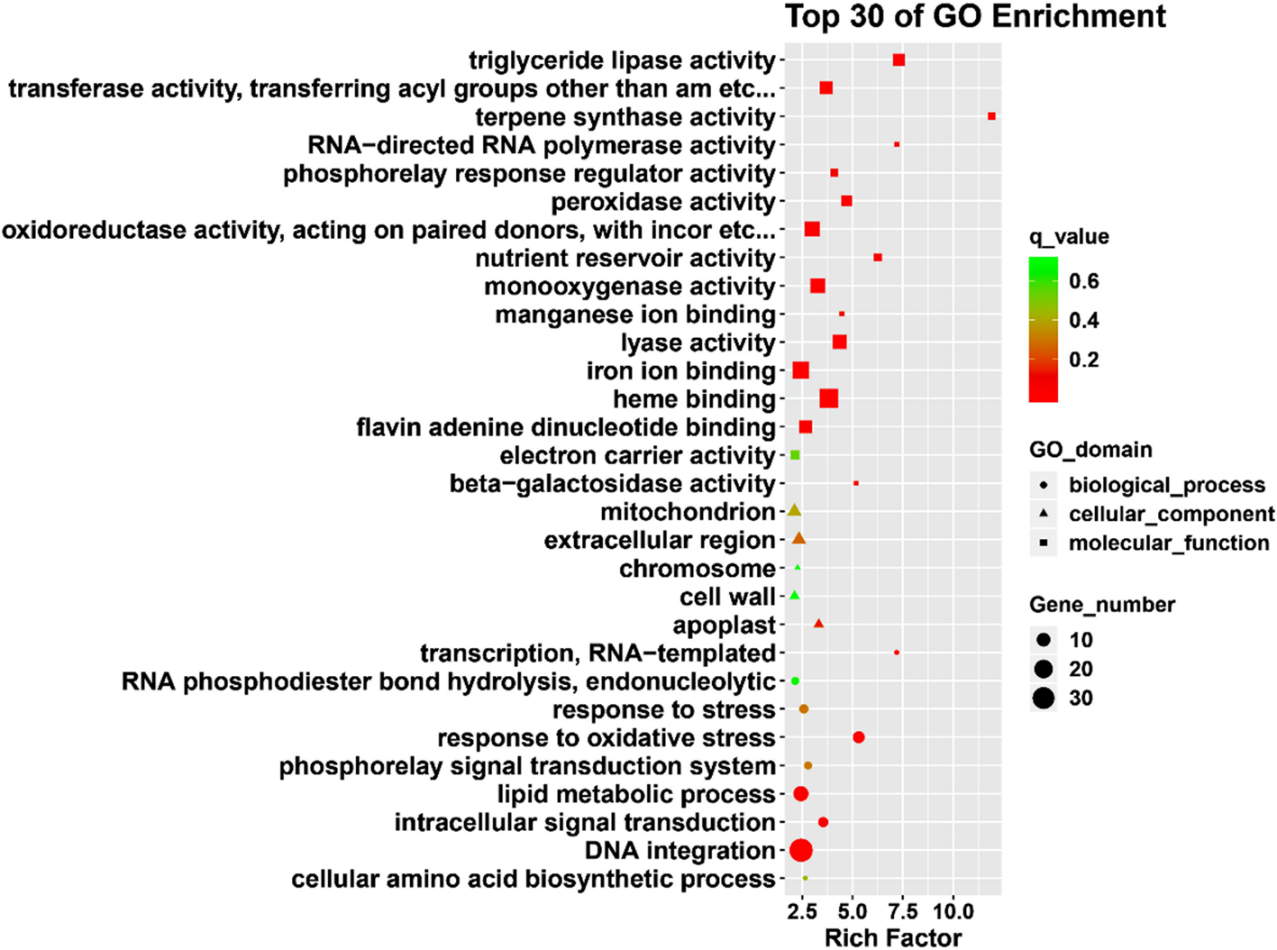
GO enrichment analysis of DEGs under high-temperature treatment. The figure shows the top 30 terms, and the size of the points indicates the number of differentially expressed genes involved in the path. The color scale indicates the significance level (q-value ≤ 0.05). The rich factor is the ratio between the number of DEGs and all genes enriched for the terms.

Comparing the 130,003 final unigenes with the Nr database, a total of 71,633 unigenes were annotated, accounting for approximately 55.1% of the total unigenes (Supplementary Table S5). To identify the DEGs significantly enriched for GO terms, the function of DEGs after high-temperature stress was analyzed using Web-based agriGO software. Among the 1,221 differentially expressed genes, 827 DEGs were significantly enriched for 56 GO terms (q-value ≤ 5%) (Supplementary Fig. S2; Table S6). In the biological process category, metabolic processes (GO: 0,008,152) (n = 72) and oxidation–reduction process (GO: 0,055,114) (n = 49) were the most abundant subcategories. In the cell components category, extracellular regions (GO: 0,005,576) (n = 8) and mitochondrion (GO: 0,005,739) (n = 6) were the most abundant subcategories. In the molecular function category, metal ion binding (GO: 0,046,872) (n = 77), zinc ion binding (GO: 0,008,270) (n = 77), and nucleic acid binding (GO: 0,003,676) (n = 69) were abundant subcategories (Fig. 4; Supplementary Table S6).

**Figure 5:**
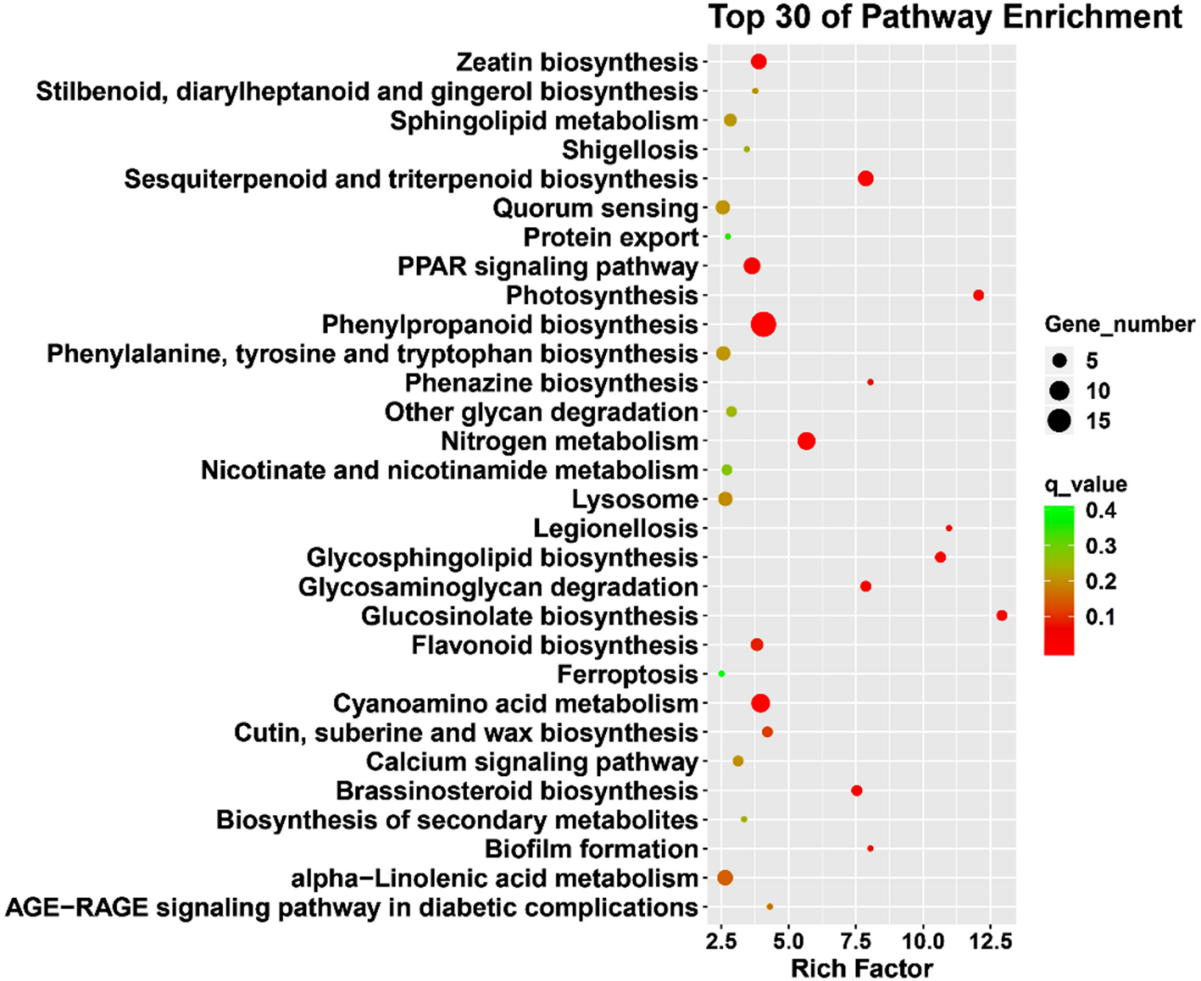
KEGG enrichment analysis of differentially expressed genes under high-temperature treatment. The figure shows the top 30 pathways. The size of the dot indicates the number of DEGs involved in the pathway. The color scale indicates the significance level (q-value ≤ 0.05). The rich factor is the ratio between the number of DEGs and all genes enriched for the pathways.

To further explore DEGs involved in the biological pathway and signal transduction pathway of *S. pohuashanensis*, the number of DEGs in each KEGG pathway was estimated. A total of 1,221 DEGs were assigned to the 151 KEGG pathways (q-value ≤ 0.05). These pathways were mainly related to the carbohydrate metabolism, biosynthesis of other secondary metabolites, lipid metabolism, energy metabolism, amino acid metabolism, transport and catabolism, endocrine system, signal transduction, metabolism of terpenoids and polyketides, folding, sorting and degradation, and metabolism of cofactors and vitamins (Fig. 5; Supplementary Table S7). According to the FPKM value of DEGs and the enrichment annotation of DEGs in go and KEGG, DEGs were screened and further analyzed according to signal transduction, transcriptional regulation, ROS homeostasis and protein homeostasis, and metabolic process.

### Differential expression analysis of genes related to signal transduction

Among the 1,221 DEGs, 41 putative genes correlated with signal transduction for high-temperature stress were identified (Supplementary Table S8). In this study, the signal transduction pathways of plant response to high-temperature stress include calcium signaling transduction, RLKs, phosphatidylinositol signaling, mitogen activated protein kinase (MAPK) signaling, cyclic adenosine monophosphate (cAMP) signaling, phospholipase D (PLD) signaling and plant hormones.

#### Calcium signaling

**Figure 6:**
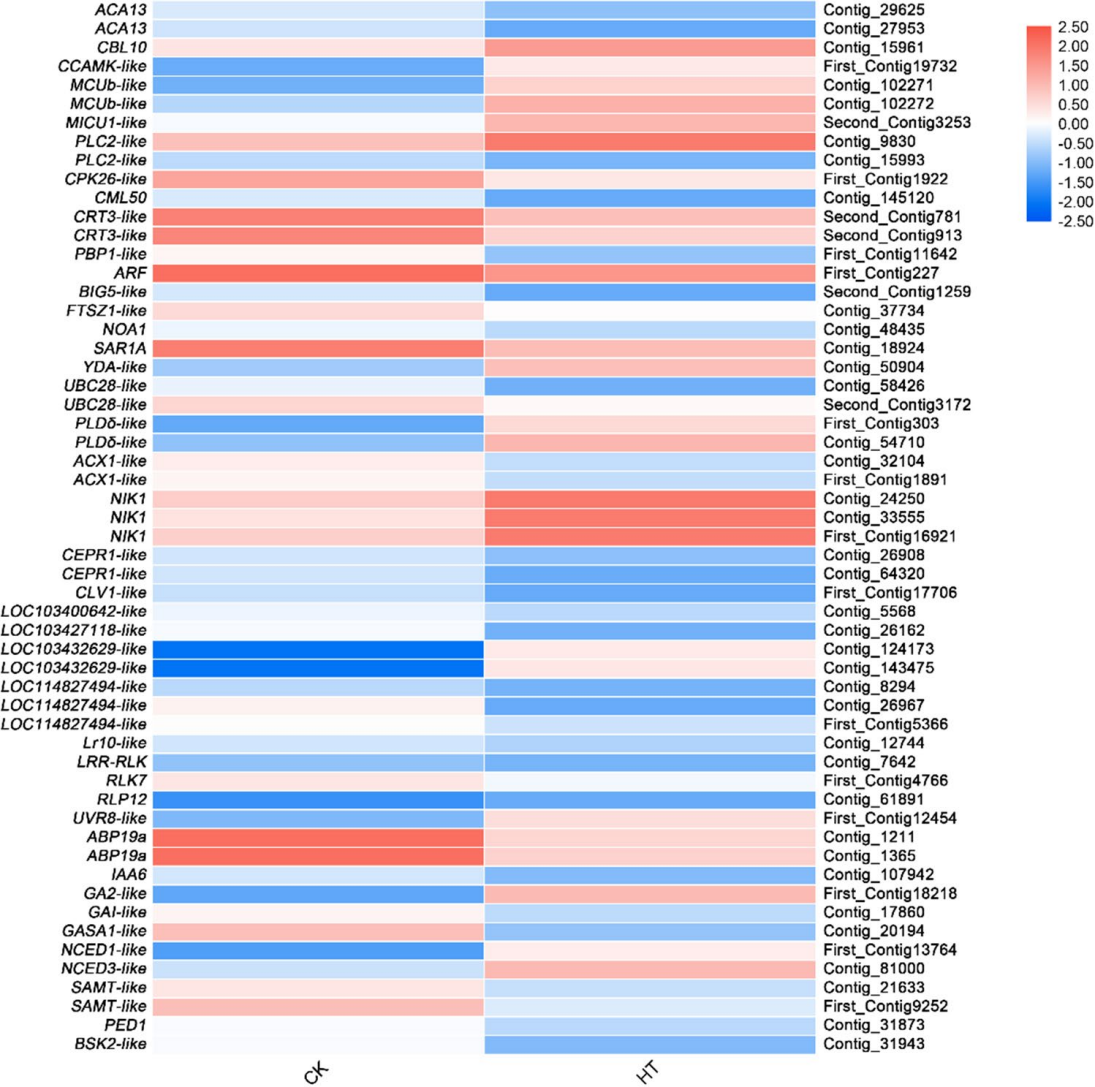
Relative expression levels of DEGs related to signal transduction pathway. The rows in the heat map represent the individual gene IDs, and the columns represent the samples taken from high-temperature treatment (HT) and control (CK). The changes in expression are indicated by colors ranging from blue (down-regulated) to yellow red (up-regulated).

After further analysis, 11 DEGs were found to be involved in the calcium signaling transduction pathway. Calcium signaling-related genes *CBL10*, *CCAMK-like* and *MCUb* were up-regulated, and *CPK26-like*, *CML50*, *PBP1-like* and G protein-related genes *ARF, BIG5-like*, *FTSZ1-like*, *NOA1*, and *SAR1A* were down-regulated under high temperature stress (Fig. 6; Supplementary Table S8).

#### Receptor-like kinase

Eleven DEGs belong to receptor-like kinase (RLK) were found, in particular, leucine-rich repeat receptor-like kinase (LRR-RLK) genes. In the LRR-RLK, there were 2 genes upregulated (*NIK1* and LOC103432629*-like*) and 9 (*CEPR1-like*, *CLV1-like*, LOC114827494*-like*, LOC103427118*-like*, LOC103400642*-like*, *Lr10-like*, *LRR-RLK*, *RLK7*, and *RLP12*) were downregulated (Fig. 6; Supplementary Table S8).

#### Intracellular signaling pathways

Moreover, 10 genes related to intracellular signaling pathways were identified. In the phosphatidylinositol signaling pathway, only *PLC2-like* was upregulated and 6 genes, *ARF*, *BIG5-like*, *FTSZ1-like*, *NOA1*, *PLC2-like*, and *SAR1A* were downregulated. In the MAPK signaling pathway, *YDA-like* was upregulated and *UBC28-like* was downregulated. In thecAMP signaling pathway, *PLDδ-like* was upregulated and *ACX1-like* was downregulated. In the PLD signaling pathway, only *PLD δ-like* was up-regulated (Fig. 6; Supplementary Table S8).

#### Plant hormone

Interestingly, 10 genes involved in plant hormone synthesis have also been identified from DEGs, *UVR8-like*, a gene related to HY5 regulation, *GA2-like* associated with gibberellin (GA) precursor synthesis, *NCED1-like*, and *NCED3-like* related to abscisic acid (ABA) synthesis were upregulated. However, *ABP19A*, *IAA6*, *GAI-like*, *GASA1-like*, *SAMT-like*, *PED1*, and *BSK2-like* were downregulated (Fig. 6; Supplementary Table S8).

### Differential expression analysis of genes related to transcription regulation

As the most important regulators in plants, HSFs play an important role in the transcriptional regulatory network of plant responses to high-temperature stress. In this study, 26 DEGs were identified related to TFs (Supplementary Table S9).

#### HSFs

**Figure 7:**
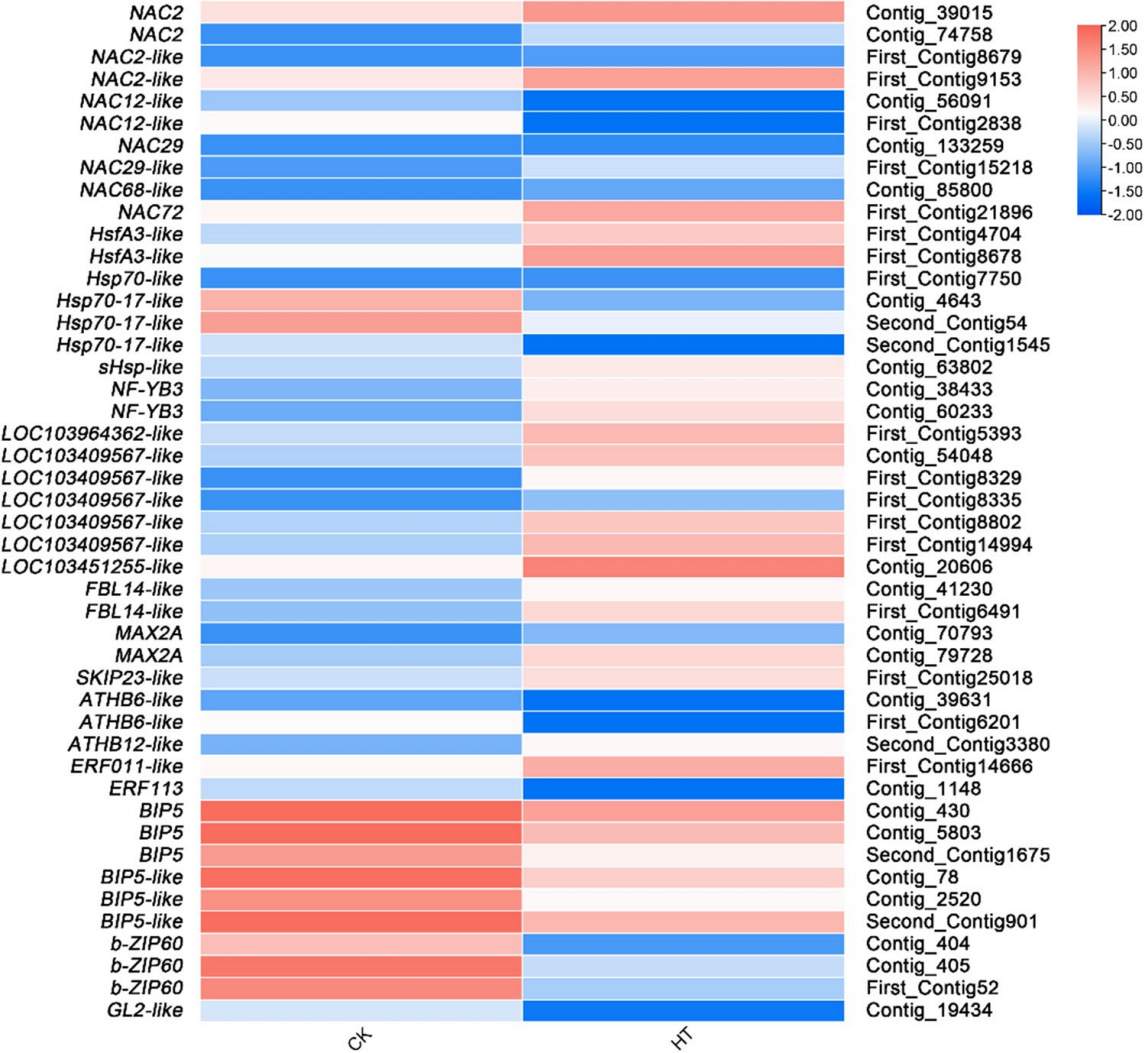
Relative expression levels of DEGs related to transcriptional regulation. The rows in the heat map represent the individual gene IDs, and the columns represent the samples taken from high-temperature treatment (HT) and control (CK). The changes in expression are indicated by colors ranging from blue (down-regulated) to yellow red (up-regulated).

After further analysis, 9 HSFs (e.g., Hsf, Hsp, BIP, bZIP, and *WRKY)* response to high temperature stress were identified. Two Hsf genes, *HsfA3-like NF-YB3*, and 2 Hsp genes, *Hsp70-like* and *sHsp-like*, were also found to be upregulated. Two down-regulated BIP genes (*BIP5* and *BIP5-like*) were found. However, only one b-ZIP gene (*bZIP60*) and one WRKY gene (*GL2-like*) appeared to be DEGs (Fig. 7, Supplementary Table S9).

#### TFs

Eleven TFs response to high temperature stress were identified. NAC genes were found to be upregulated (*NAC2*, *NAC29*, *NAC2-like*, *NAC29-like*, *NAC68-like*, and *Nac72*) and one (*NAC12-like*) was downregulated. One upregulated ATHB gene (*ATHB12-like*), one down-regulated ATHB gene *(ATHB6-like*), one upregulated ERF gene (*ERF011-like*)*,* and one down-regulated ERF gene *(ERF113*) were found. (Fig. 7, Supplementary Table S9).

#### F-box

Six genes of F-box protein, a key component of the E3 ubiquitin ligase SCF complex, were identified in DEGs. Three upregulated F-box/kelch repeat protein genes (LOC103964362*-like*, LOC103409567*-like*, and LOC103451255*-like*), 2 upregulated F-box/LRR-repeat protein genes (*FBL14-like* and *MAX2A*)*,* and F-box protein gene *(SKIP23-like*) were identified (Fig. 7, Supplementary Table S9).

### Differential expression analysis of genes related to protein homeostasis and ROS homeostasis

**Figure 8:**
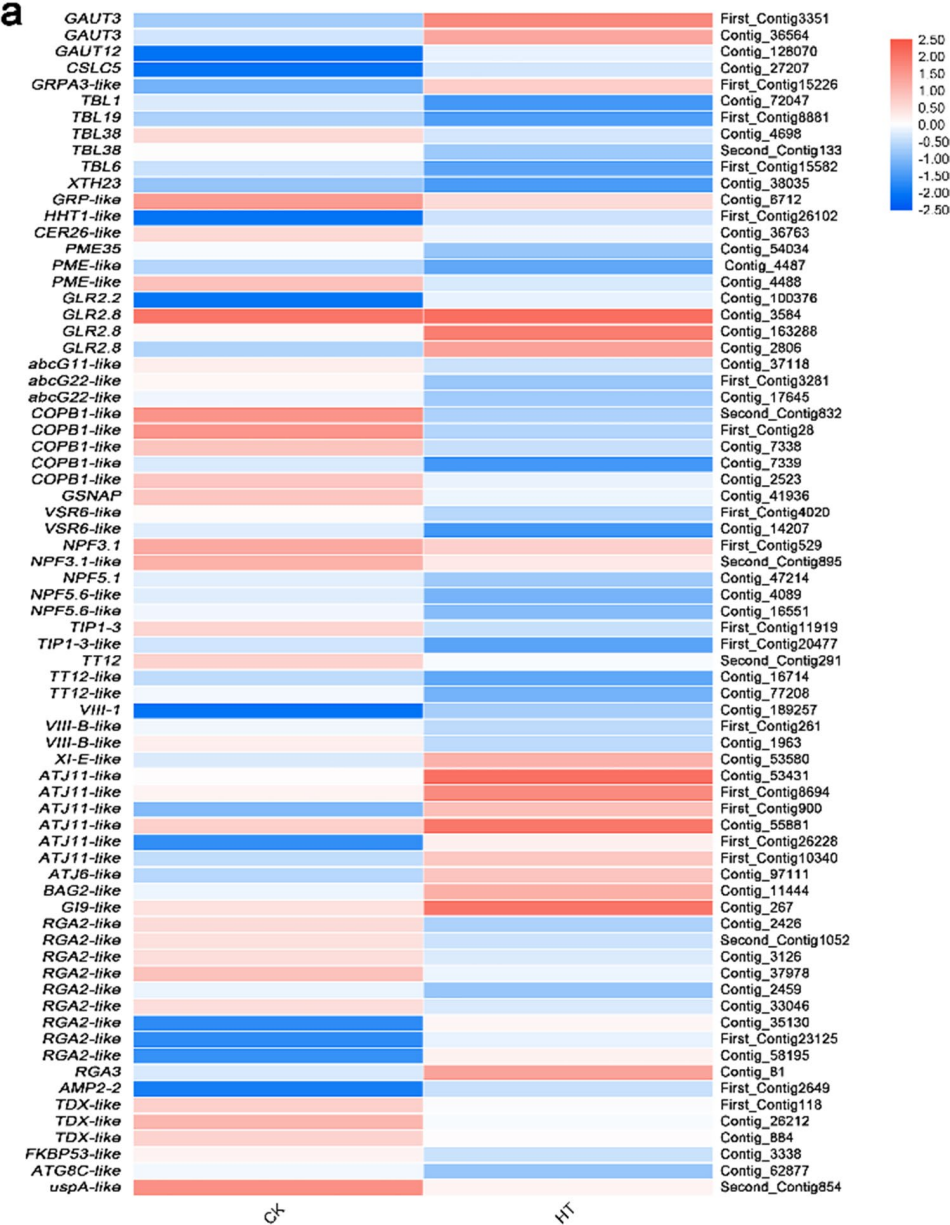

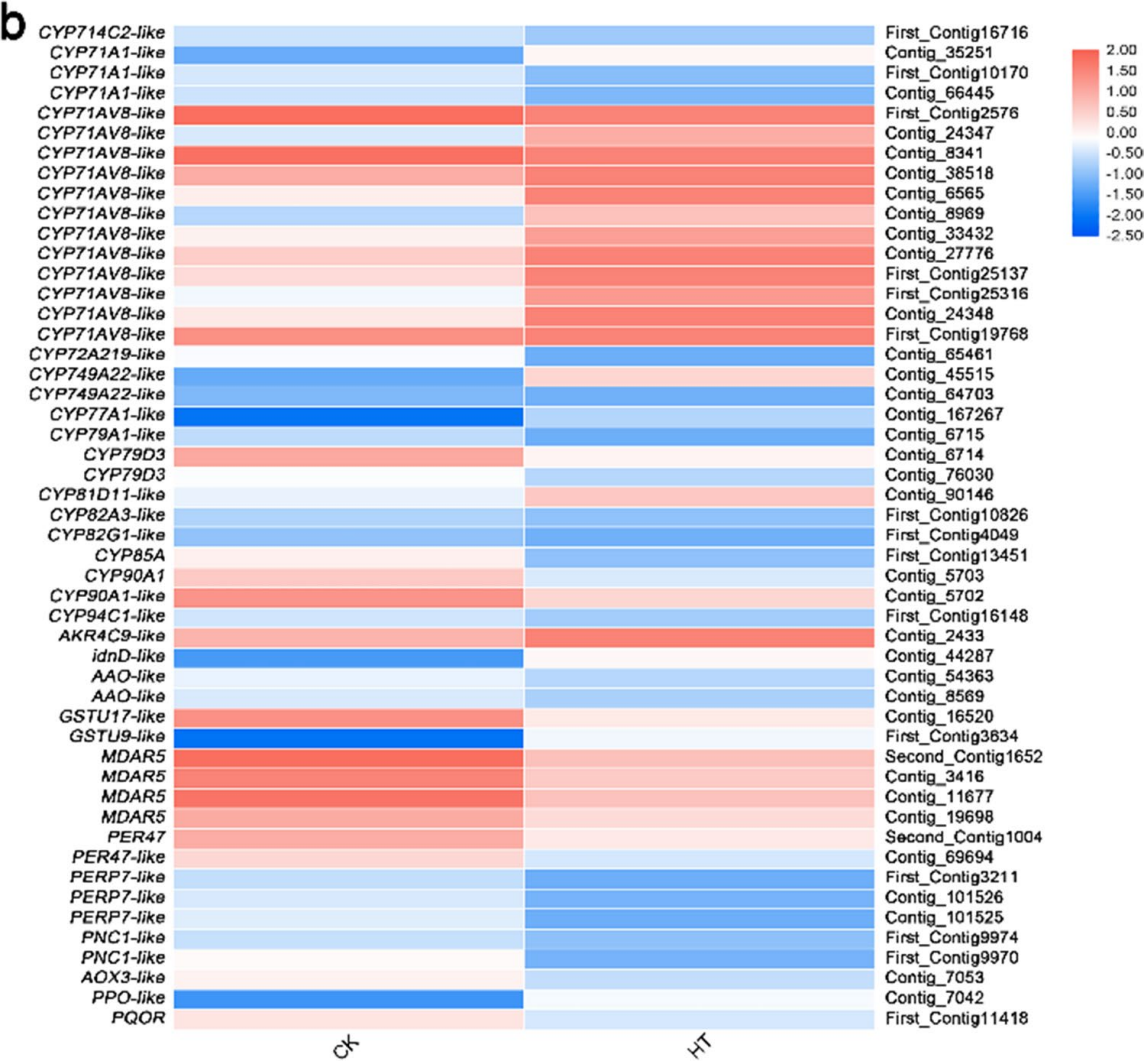
Relative expression levels of DEGs related to protein homeostasis (**a**) and ROS homeostasis (**b**). The rows in the heat map represent the individual gene IDs, and the columns represent the samples taken from high-temperature treatment (HT) and control (CK). The changes in expression are indicated by colors ranging from blue (down-regulated) to yellow red (up-regulated).

In this study, 70 putative genes correlated with protein homeostasis and ROS homeostasis for high-temperature stress were identified (Fig. 8, Supplementary Table S10), which mainly involved cell wall, early secretary pathway, active transport of plasma membrane, chaperone, autophagy, detoxification, enzymatic antioxidant system.

#### Cell wall

Fourteen DEGs were found to be involved in the cell wall synthesis and modification and 5 were up-regulated, G*AUT3*, *GRPA3-like* and *HHT1-like*, while 9 were down-regulated *CER26-like*, *GRP-like*, *PME35*, *PME-like*, *TBL1*, *TBL6*, *TBL19*, *TBL38* and *XTH23*(Fig. 8, Supplementary Table S10).

#### Plasma membrane and early secretary pathway

In the plasma membrane and transmembrane transport, 18 DEGs were identified. Glutamate receptor protein (GLR) gene *GLR2.2* and *GLR2.8* were up-regulated. Dynein genes *XI-E-like* and *VIII-1* were up-regulated; aquaporin genes *TIP1-3* and *TIP1-3-like*, transporter genes *abcG11-like*, *abcG22-like*, *TT12*, *TT12-like*, *NPF3.1*, *NPF3.1-like*, *NPF5.1*and *NPF5.6*, coatomer subunit gene *COPB1-like*, ER-GA-transported protein complex gene *SAR1A*, vacuolar transport-related genes *GSNAP* and *VSR6-like*, endocytosis-related gene *EHD1-like*, dynein gene *VIII-E-like*, galacturonidase-related gene *LUG-like* were down-regulated (Fig. 8, Supplementary Table S10).

#### Chaperone and autophagy

In addition to protein homeostasis-related genes, 12 DEGs were identified, chaperone DnaJ genes *ATJ11-like* and *ATJ6-like*, Bcl-2-associated athano gene *BAG2-like*, disease resistance protein genes *RGA2-like* and *RGA3* and vicilin gene *AMP2-2* were up-regulated and disease resistance protein gene *RGA2-like*, autophagy gene *ATG8C* and universal stress protein gene *uspA-like* were down-regulated (Fig. 8, Supplementary Table S10).

#### CYP450

DEGs related to ROS homeostasis were also identified. In the detoxification, 7 genes, *CYP71A1-like*, *CYP71AV8-like*, *CYP749A22-like*, *CYP77A1-like*, *CYP81D11-like*, *AKR4C9-like* and *idnD-like* were up-regulated and 11 genes, *CYP714C2-like*, *CYP71A1-like*, *CYP72A219-like*, *CYP79A1-like*, *CYP79D3*, *CYP82A3-like*, *CYP82G1-like*, *CYP85A*, *CYP90A1*, *CYP90A1-like* and *CYP94C1-like* were down-regulated (Fig. 8, Supplementary Table 10).

#### ROS scavenger enzyme

In the ROS scavenger enzyme-related genes, only *GSTU9-like* was up-regulated and 7 genes, *GSTU17-like*, *MDAR5*, *PER47*, *PER47-like*, *PERP7-like* and *PNC1-like* were down-regulated (Fig. 8, Supplementary Table S10).

### Differential expression analysis of genes related to metabolism process

**Figure 9:**
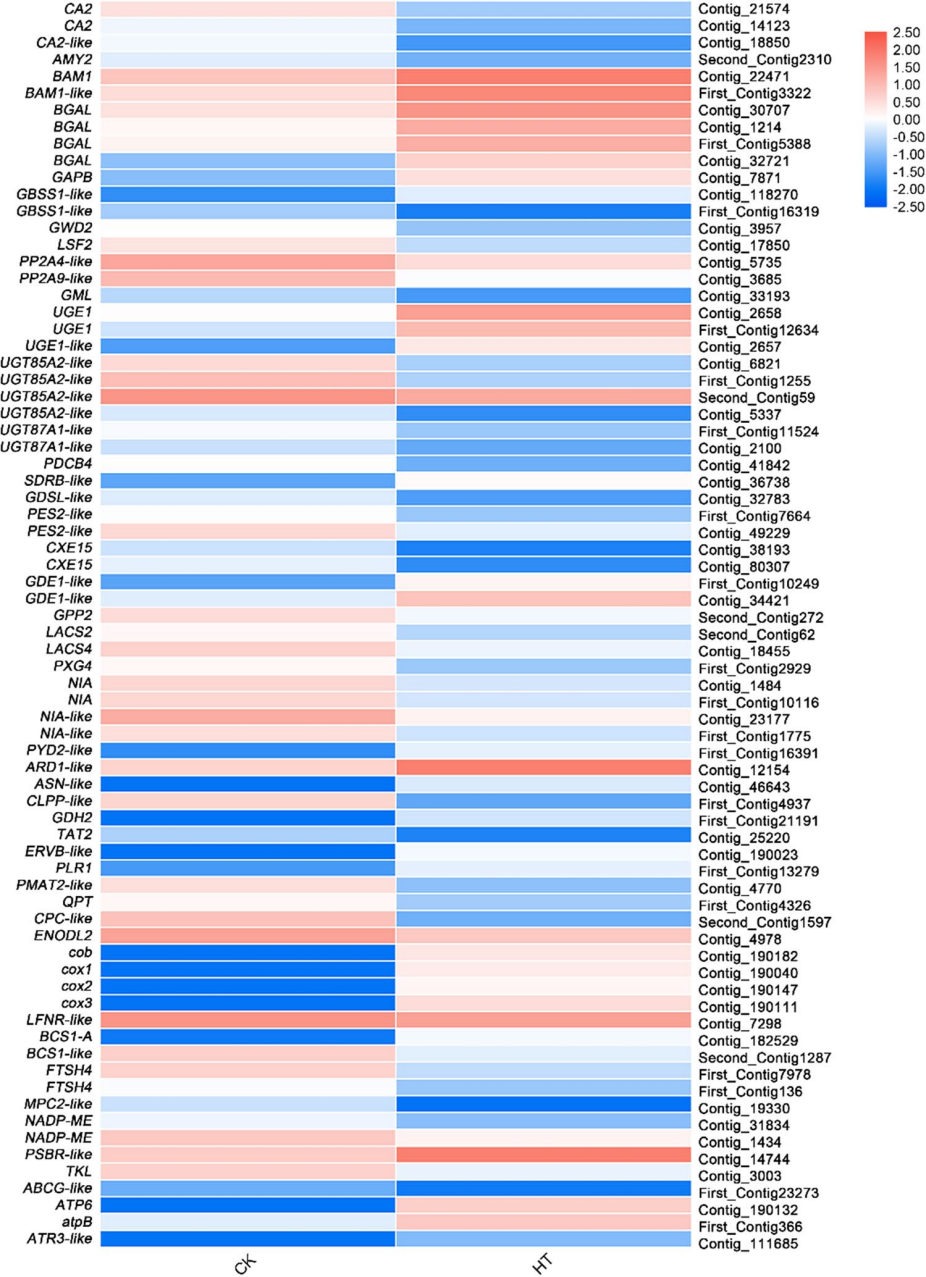
Relative expression levels of DEGs related to metabolism process. The rows in the heat map represent the individual gene IDs, and the columns represent the samples taken from high-temperature treatment (HT) and control (CK). The changes in expression are indicated by colors ranging from blue (down-regulated) to yellow red (up-regulated).

In this study, 58 genes related to material and energy metabolic processes in DEGs were identified. mainly involving carbohydrate metabolism, lipid metabolism, amino acid metabolism, photosynthesis, respiration (Fig. 9, Supplementary Table S11).

### Carbohydrate metabolism

Seventeen genes related to carbohydrate metabolism were identified, and 7 genes, *BAM1*, *BAM1-like*, *BGAL*, *GAPB*, *GBSS1-like*, *UGE1* and *UGE1-like* were up-regulated, while 10 genes, *AMY2*, *GBSS1-like*, *GWD2*, *LSF2*, *PP2A4-like*, *PP2A9-like*, *GML*, *UGT85A2-like*, *UGT87A1-like* and *PDCB4* were down-regulated (Fig. 9, Supplementary Table S11).

#### Lipid metabolism

In lipid metabolism, 9 genes were identified from DEGs and *SDRB-like* and *GDE1-like* were up-regulated, while 7 genes, *GDSL-like*, *PES2-like*, *CXE15*, *GPP2*, *LACS2*, *LACS4* and *PXG4* were down-regulated (Fig. 9, Supplementary Table S11).

#### Nitrogen metabolism and amino acid metabolism

In nitrogen metabolism and amino acid metabolism, 9 genes were also detected from DEGs, and 5 genes, *PYD2-like*, *ARD1-like*, *ASN-like*, *GDH2* and *ERVB-like* were up-regulated and 4 genes, *NIA*, *NIA-like*, *CLPP-like* and *TAT2* were down-regulated (Fig. 9, Supplementary Table S11).

#### Energy metabolism

Eighteen genes related to energy metabolism were also identified from DEGs. Seven genes related to electron transport chain were found and four respiratory electron transport chain genes, *cob*, *cox1*, *cox2*, *cox3* were up-regulated, while only one photosynthetic electron transport chain gene, *LFNR-like* was down-regulated. In respiration, entire 4 genes, *BCS1-A*, *BCS1-like*, *FTSH4*, *MPC2-like* were down-regulated. In photosynthesis, only *PSBR-like* was up-regulated and 2 genes, *NADP-ME* and *TKL* were down-regulated (Fig. 9, Supplementary Table S11).

### Quantitative RT-PCR determination of relative DEGs

**Figure 10:**
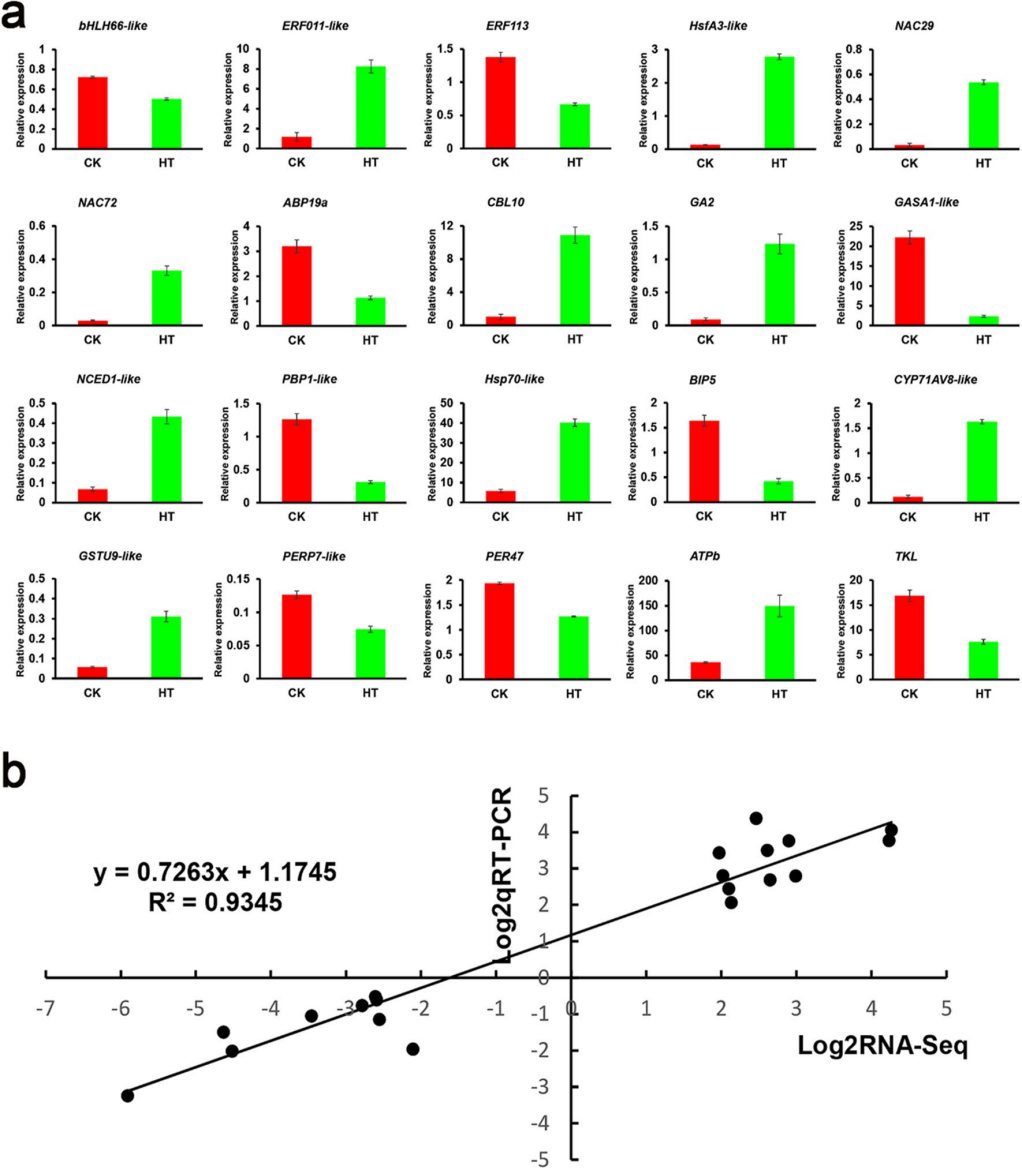
Relative gene expression of DEGs analyzed by quantitative RT-PCR in response to high-temperature stress treatment. (**a**) quantitative RT-PCR data were normalized using the *S. pohuashanensis* Actin β gene and are shown relative to CK (normal condition). X-axes show different treatments (CK, normal condition and HT, high temperature condition) and Y-axes are scales of relative expression level (error bars indicate SD). (**b**) Correlation of expression levels between RNA-seq and qRT-PCR that identified 20 HT vs. CK. The log_2_qRT-PCR (y-axis) was plotted against log_2_RNA-seq (x-axis).

In order to verify the accuracy of the transcriptome sequencing results, 20 genes were randomly selected for quantitative RT-PCR verification (Fig. 10a). The results showed that the relative expression patterns of these genes in the HT and CK groups were consistent with those obtained by RNA-Seq (Supplementary Table S4). In addition, we determined their response functions at high temperatures. Comparison of transcriptomic data and qRT-PCR results showed a high correlation (R^2^ = 0.9345) (Fig. 10b).

## Discussion

Temperature factor is one of the most important factors which can affect the growth and development of plants and limit the geographical distribution of plants^38^. It is particularly important to explore the molecular mechanism of plant response to high temperature during introduction and acclimatization. Based on Illumina HiSeq2000 platform, this study conducted RNA-seq on leaves of *S. pohuashanensis*, and obtained high-quality transcriptome data responding to high-temperature stress. A total of 1,221 DEGs were obtained, among which 752 were up-regulated and 469 were down-regulated. DEGs were mainly concentrated in calcium signal transduction, RLKs, TFs, cell wall and membrane system, chaperone, ROS and metabolism, indicating that high-temperature stress has a multi-aspect effect on life activities of *S. pohuashanensis*.

The signal transduction pathways of plant response to high-temperature stress include calcium signaling transduction, phosphatidyl inositol signaling, MAPK signaling, cAMP signaling, PLD signaling, RLKs, and plant hormone signal transduction^22^. High-temperature stress could affected the transport of Ca^2+^ on the plasma membrane of *S. pohuashanensis* and promotes the function of G proteins/small G proteins. The increase in intracellular Ca^2+^ concentration activates the G-protein-mediated phosphatidyl inositol signaling pathway (G-protein-PLC-PKC)^39^. PLC located on the cell membrane can combine with SA to participate in the response to high-temperature stress, and its activity reaches the maximum after 40 min of high-temperature treatment in *Pisum sativum*^40,41^. In this study, *PLC2-like* was upregulated (Supplementary Table S4; Table S8), indicating that PLC might play a specific role in the response to high-temperature stress in *S. pohuashanensis*. When ligands (hormones, neurotransmitters, and growth factors, etc.) bind to specific receptors on the cell membrane, adenylate cyclase is activated through G protein-coupled receptors to increase the production of cAMP. Then, cAMP-activated protein kinase A (PKA). PKA regulates the activities of various proteins, including TFs, through phosphorylation, so as to regulate a variety of physiological molecules in cells such as ion channels, cytoskeleton proteins, and enzymes^42^. *PLDδ* may regulate signal transduction in the process of plant stress resistance through phosphatidic acid (PA), an important signaling molecule in plant cell signal transduction^43^. PLD can also directly interact with heterotrimer G protein to influence cell signal transduction^44^. Moreover, we found that genes related to intercellular and intracellular signaling pathways, MAPK signaling pathways (*YDA-like*), cAMP signaling pathways*,* and PLD signaling pathways *(PLDδ*) were up-regulated (Supplementary Table S4, Table S9), which implies that *S. pohuashanensis* could transports high-temperature signals to the nucleus through the MAPK, cAMP, and PLD pathways, further activating hormone signaling and TF regulation.

In our study, HSFs (*HsfA3-like*, *BIP5*, *BIP5-like*, *bZIP60*, *GL2-like*, *sHsp-like*, *Hsp70-like* and *Hsp70-17-like*) and other TF (*ATHB6-like*, *ATHB12-like*, *ERF011-like*, *ERF113*) genes were found in the high-temperature transcriptome of *S. pohuashanensis* (Supplementary Table S4; Table S9). High-temperature stress causes damage to membrane proteins, denaturation and inactivation of various enzymes, and accumulation of ROS leading to cell injury and death. *Hsfs* function as transcriptional activators for several genes including Hsps, regulating the expression of Hsps to maintain homeostasis in plants against heat and chemical stresses^45^. The expression of Hsps by Hsfs genes is regulated via their interactions with a palindromic binding motif in the promoter region of heat-responsive genes, such as heat shock elements to counteract heat stress-induced ROS^46^. Class A Hsfs function as central activators of the HSR, and HsfAs can activate the expression of HSR genes and regulate the synthesis of chaperones and enzymes involved in unfolded protein degradation and ROS scavenging^47^. Under normal and long-term heat stress conditions, they are strictly regulated by various mechanisms to avoid detrimental effects upon activation or over-accumulation^18^. In a recent report, both *LlHSFA3A* and *LlHSFA3B* of *Lilium spp.* are induced by heat stress. Overexpressing *LlHsfA3A* in Arabidopsis enhanced its basal and acquired thermotolerance, while overexpressing *LlHsfA3B* just enhanced its acquired thermotolerance^48^. Furthermore, *AtHsfA3* was remarkably promoted in *AtDREB2A CA* and *AtDREB2C* overexpression seedlings, and the downregulation of *Hsp18.1-CI* and *Hsp25.3-P* in *dreb2a* and *dreb2c* depends on *AtHsfA3*^49,50^. Li et al. confirmed that the direct interaction between *AtHsfA2* and *AtHsfA3* through genetic analysis, gene expression analysis and yeast two-hybrid assays^51^. *HsfA3* regulates expression of many heat-inducible genes in the transcriptional cascade downstream of the DREB2A stress-regulatory system and functions in acquisition of thermotolerance under the control of the DREB2A cascade^52^. In addition, in the *Phoenix dactylifera*, transcript accumulation of *PdHsfA3* imparting protection from heat-induced ROS generation and boosting the antioxidative response^53^. *HsfA3* might play a similar role in the regulatory network in response to high-temperature stress in *S. pohuashanensis*. By functioning as molecular chaperones, Hsps prevent protein denaturation and aggregation. Hsp70 is the most prominent Hsp molecule that is visible when environmental temperature increment or other stresses that damage proteins^54^. In general, the expression of the *Hsp70* positively correlates with the ability to tolerate high-temperature stress^55^. In the event of stress, Hsp70 provides protection to the cells by refolding and disaggregating substrate proteins^56^. A group of sHsps protect photosynthetic machinery from denaturation. sHsp21 plays an essential role in the development of thermomemory. It is reported that, in the tomato, these SlHsp21 mostly involve in the protection of PSII under high-temperature stress^57^. Transgenic Arabidopsis plants overexpressing Hsp21 show high tolerance to HS^58^. Other HSFs, like WRKYs, are another regulatory factor in response to high-temperature stress. *AtWRKY25* can increase the contents of *HsfA2*, *HsfB1*, *HsfB2A,* and *Hsp100F* in *AtWRKY25* overexpression lines of *A. thaliana*, which significantly improves the high-temperature resistance of the plants^59,60^. New evidence suggests that other TFs except for HSFs, for example ATHB and ERFs were also regulating plant's thermotolerance^61^. ATHB6 is a specific TF of higher plants and one of the components of the early signal transduction pathway, participating in the ABA signaling pathway as a main specific switch^62^. Moreover, ATHB6, ATHB7, and ATHB12 may interact with each other by forming heterodimers, which can activate potential complexes in plants^63^. ERFs belong to the AP2/ERF superfamily, which regulate the growth, development, and stress response of plants throughout the life cycle^64^. ERFs are early response factors that influence the expression of jasmonic acid (JA), salicylic acid (SA), ethylene (ETH), H_2_O_2_, and other signals by affecting the expression of downstream negative regulators, which affects the expression of defense genes and assists plants to resist environmental stress^65,66^. The increased transcription of *MaERF1* in bananas causes the accumulation of MaERF1 under high temperature, indicating that MaERF1 may play an important role in the activation of the heat defense system^67^. Transcriptional regulations would play important roles in the regulatory network of *S. pohuashanensis* in response to high-temperature stress.

Hyperthermia breaks the homeostasis of ROS in cells, and changes of membrane proteins and membrane lipids, resulting in increased cell permeability, loss of cell membrane integrity and electrolyte outflow^68^. In this study, it was found that the content of MDA in *S. pohuashanensis* leaves increased significantly under high temperature stress. MDA is often used as an important indicator to reflect membrane lipid peroxidation, and its concentration represents the intensity of membrane lipid peroxidation and the damage degree of membrane system^69^. In addition, we found that *GLR2.2* and *GLR2.8* were up-regulated (Supplementary Table S10). Glutamate receptor protein (GLR) is a plant injury receptor, which can induce the increase of intracellular Ca^2+^ concentration in plants, and convert this signal into intracellular signal response to injury stimulation to induce plant defense response^70^. The increase of MDA content and the up regulation of GLRS in leaves of *S. pohuashanensis* under high temperature stress indicated that the cell membrane structure of *S. pohuashanensis* was damaged and could not maintain the normal cell structure. In addition, the contents of Pro, soluble sugar and other osmoregulation substances in *S. pohuashanensis* leaves also increased significantly after high temperature stress, indicating that the permeability of *S. pohuashanensis* cells increased and the normal metabolic activities of cells were affected. Transmembrane transport is one of the cellular activities affected by high temperature stress in *S. pohuashanensis*. Members of the plant NPF family have the function of transporting nitrate and small peptides, and several other NPF substrates, e.g. indole acetic acid (IAA), ABA, JAs, GAs, have been identified in recent years^71,72^. Meanwhile, the early secretion pathway of leaf cells was affected under high temperature stress. The early secretory pathway is an important stage for quality control and sorting of proteins^73^. Protein vesicles are formed at the endoplasmic reticulum export sites (ERES) and coat protein complex II (COPII) after preliminary processing of proteins in endoplasmic reticulum (ER), and separated from ER^38^. The above results showed that the cellular structures of the leaves of *S. pohuashanensis* were also destroyed due to the change of osmotic potential, and the transport capacity of transporters and plasma membrane (PM) decreased. It was unable to synthesize enough hormones, ROS scavenging enzymes and other substances to metabolize toxic substances significantly to enhance the further resistance of *S. pohuashanensis* under high temperature stress.

However, there are still some mechanisms of action for the maintenance of protein homeostasis under high temperature stress. In this study, specific expressions of molecular chaperones, resistant proteins and other types of genes were found (Supplementary Table S10). High temperatures lead to the accumulation of unfolded proteins in plant cells, which must be regenerated or degraded by plants in order to maintain normal life activities. Molecular chaperones are a class of proteins that correctly fold, assemble, transport other proteins and mediate the degradation of misfolded proteins, reducing the damage caused by stress to cells^74^. For instance, Heat shock protein 70 (Hsp70), a molecular chaperone, plays an extremely important role in biotic and abiotic stresses, and its activity is regulated by DnaJ, which is another type of chaperone. DnaJ can enhance the affinity of Hsp70 with its substrates, promote the release of Hsp70^75,76^. Up-regulated expression of chaperone genes indicated that *S. pohuashanensis* could regulates its homeostasis and reduces its damage by high-temperature to recombining various proteins denatured under high-temperature stress. Furthermore, in this study, autophagy related gene *ATG8C-like* and universal stress protein gene *USP-like* were down-regulated, and resistance genes *RGA2-like*, *RGA3* and vicilin gene *AMP2-2* were up-regulated. Autophagy is an intracellular quality control system that removes non-functional proteins and damaged cell components, and this homeostatic pathway is important for the energy balance of plant cells^77^. Recent studies have shown that autophagy is involved in the response of plants to high-temperature stress, and the impaired autophagy function of *A. thaliana* and *Solanum lycopersicum* leads to the accumulation of aggregated proteins, resulting in the decline of their heat tolerance^78,79^. High-temperature significantly induced up-regulated expression of autophagy-related genes ATG genes *SlATG5* and *SLATG7* in tomato, which resisted high temperature injury by protecting membrane structure and photosynthetic system^80^. Besides, some resistance proteins e.g. resistance gene analogs (RGAs), vicilin and universal stress proteins(USPs) seem to play a role in maintaining protein homeostasis of *S. pohuashanensis* under high-temperature stress^42,81^.

ROS is produced quickly after the beginning of high temperature stress, and can be used as an early messenger to activate stress response, and plants acquire heat tolerance by activating ROS scavenging system^82^. In this study, the activity of ROS scavenging enzymes of *S. pohuashanensis* showed that the activities of POD, T-SOD and APX in leaves of *S. pohuashanensis* were decreased after 8 h at 43℃. RNA-seq results showed that genes related to ROS scavenging enzymes in DEGs, e.g. *PER47*, *PER47-like*, *PERP7-like* and *PNC1-like* were down-regulated (Supplementary Table S10), which was consistent with the results of physiological indicators. Melakeselam and Zhou found that high temperature decreased POD and SOD activities in rape leaves, but had little effect on CAT activity^83^. APXs and CATs are considered to be two kinds of ROS scavenging enzymes necessary for ROS detoxification^23^. Although no genes related to CAT synthesis and metabolism were found in DEGs in this study, the results of physiological indicators also showed that CAT activity was not affected by high-temperature stress. It is possible that the response of CATs to environmental stress is comprehensive rather than only response to heat stress. GSTU belongs to glutathione S-transferases (GSTs). They are involved in phytochrome phyA mediated photomorphogenesis and integration of plant hormone signals, and regulate all aspects of plant development by affecting glutathione pool^84^. Stress treatments were applied to different spike weights of *Oryza sativa* at different temperatures. The activities of POD, SOD and CAT in leaves increased with increasing treatment temperature below 29 °C. In some varieties, POD activity started to decrease at 32 °C, and all enzyme activities showed a substantial decrease when the temperature reached 39 °C^85^. The indicators of antioxidant capacity of the *Asparagus schoberioides* wild type began to decline under high temperature stress at 32 °C, while the good variety ‘981′ maintained high antioxidant activity and the heat tolerant variety ‘07–2′ showed enhanced antioxidant activity capacity, with a significantly higher increase in the activities of some ROS scavenging enzymes (POD, GSH-Px) activity was significantly higher than wild type and '981′ under 28 °C treatment, indicating an important correlation between ROS scavenging enzymes activity and heat tolerance in *A.schoberioides*^86^. The biomass accumulation and cellulose content of the double-gene transformed Arabidopsis were higher under salt stress when compared to the wild-type and single-gene transformed plants, obtained by transfecting PaSOD and RaAPX simultaneously. The cellulose content was 60–100% higher in the double-transformed and PaSOD-transformed strains and 40% higher in the RaAPX-transformed strains compared to the wild-type strain. In addition, PaSOD and RaAPX double-transformed Arabidopsis accumulated more phenolics compared to the wild type^87^. All these results indicate that the scavenging of excess ROS is an important aspect of plant response to heat stress. However, in a previous study by Peng Song et al. it was found that SOD, and then POD activities of 1-year-old seedlings of *S. pohuashanensis* under high temperature stress at 40 °C increased and then decreased^34^. Furthermore, the same results were obtained in our subsequent replicate experiments. A possible important reason for the inability of rowan trees to adapt to high temperature stress is their reduced ability to scavenge reactive oxygen species. In addition to the ROS scavenging system, detoxification also played an important role in the occurrence of high-temperature stress. The expression of *CYP71AV8-like* was up-regulated in the DEGs (Supplementary Table S10). It is known that CYP71AV8 can convert n-pentene to n-pentanone, and participate in the biosynthesis of sesquiterpene lactone and artemisinic acid in chicory^88^. CYP71AV8 belongs to cytochrome P450 (CYP450), which exists in the membrane of PM, mitochondria, Golgi apparatus, peroxisome, nuclear membrane and other organelles. It has a detoxification effect. It can usually metabolize fat soluble toxic substances into water-soluble substances, so that toxic substances can be discharged from the body. As a terminal oxygenase, CYP450 is also involved in steroid hormone synthesis and other processes in vivo^89^. In this study, 14 genes of CYP450 in DEGs were identified except for *CYP71AV8-like* (Supplementary Table S10), which indicates that CYP450 may play a role in detoxification of plant cells in *S. pohuashanensis* under high-temperature stress. These results suggest that the resistance of *S. pohuashanensis* to high-temperature stress may be caused by the cooperation of multiple defense mechanisms.

When plants are subjected to stress conditions, maintaining adequate energy metabolism is essential for effective adaptation to stress^90^. Thylakoid membrane and matrix fluidity in chloroplasts increase, leading to inactivation of various enzymes involved in photosynthesis and disruption of the membrane system under high-temperature stress^91^. High temperature stress destroys the catalytic enzymes of dark reactions at first, and then PS II of light reactions^92^. However, as stress progresses, the degree of thylakoid destruction is aggravated, and at the same time, the PSII located on the thylakoid membrane is destroyed and the opening of the PSII reaction center is reduced^34^. This is consistent with the results that the fluorescence kinetic parameters F_0_/F_m_ of the leaves of *S. pohuashanensis* did not decrease significantly before 2 h of high-temperature stress, while parameters e.g. PSII and NPQ began to decrease after 2 h of high-temperature stress. In addition, the chloroplast NADP reductase gene *LFNR-like*, malate dehydrogenase gene *NADP-ME*, and transketolase gene *TKL* in DEGs were downregulated (Supplementary Table S11), indicating that the dark reaction process of photosynthesis was affected under high temperature stress. In the respiration, cytochrome C oxidase genes *cob*, *cox1*, *cox2* and *cox3* involved in the respiratory electron transport chain were up-regulated, and mitochondrial chaperone gene *BCS1-like*, respiratory-related gene *FTSH4* and pyruvate metabolism gene *MPC2-like* were down-regulated (Supplementary Table S11), indicating that the respiration of the leaf cells of *S. pohuashanensis* was affected. It is worth mentioning that glyceraldehyde-3 phosphate dehydrogenase (GAPDH) gene GPP2 in DEGs was down-regulated (Supplementary Table S11), a substance considered to protect cells from environmental heat stress^93^. Moreover, KEGG enrichment of DEGs showed that the genes related to sulfur and nitrogen metabolism were down-regulated, tryptophan metabolism, arginine biosynthesis, phenylalanine metabolism, histidine metabolism, arginine and Pro metabolism and tyrosine metabolism were down-regulated in amino acid metabolism, indicating that the synthesis process of protein was significantly affected under high-temperature stress. The above results showed that high-temperature stress had an adverse effect on both material and energy metabolism of *S. pohuashanensis*.

**Figure 11:**
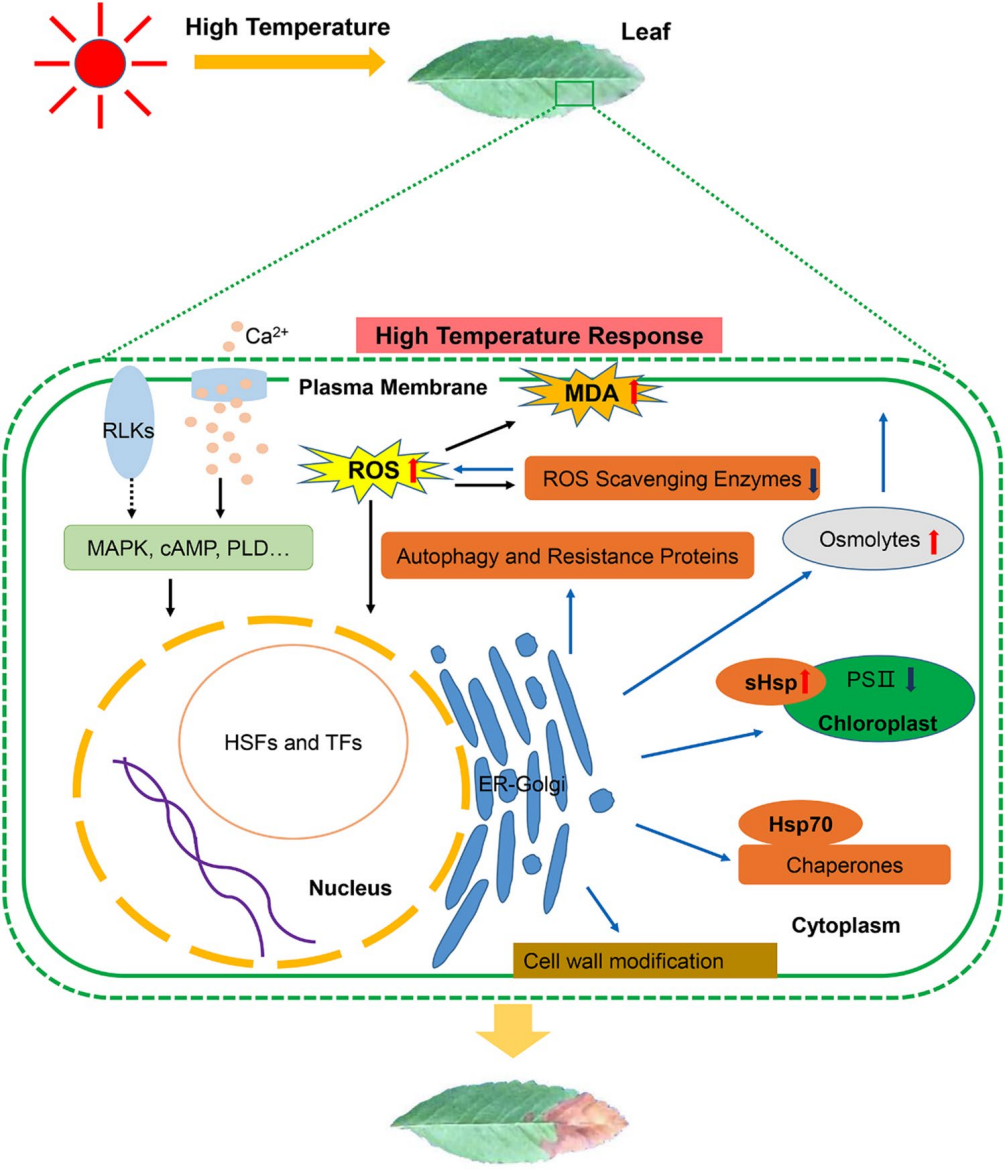
Schematic representation of the response mechanism of *S. pohuashanensis* to high temperature stress (Refer to Nishad et al. (2020) and Wang et al. (2020))^20,22^. High-temperature stress causes damage to membrane proteins, denaturation and inactivation of various enzymes, and accumulation of reactive oxygen species. High-temperature stress changes membrane fluidity, which may be sensed by proteins, such as Ca^2+^ channels and receptor-like kinases, localized at the plasma membrane. Calcium signaling plays critical roles in sensing sudden changes in temperature and activating cascades of signaling, leading to the production of Hsps that keep protein-unfolding under control. HSFs are the transcription factors that read the activation of thermosensors and induce the expression of HSPs. *HsfAs* are activated by high-temperature, and they target downstream transcription factors (such as *HsfA3*) to induce the expression of heat stress-responsive genes (*Hsp70*, *sHsp*, and other chaperones), which are important for ROS scavenging and protein homeostasis. In addition, some TF genes, including *ERFs*, *bZIPs* and *ATHBs* also play a role in the molecular mechanism of high-temperature stress in *S. pohuashanensis*. Accumulation of ROS in plants activates HSFs, which in turn activate ROS scavenging and detoxifying enzymes like APX and SOD. ROS level occurs due to the change by production of antioxidants, osmolytes, and Hsps. The major stress-responsive osmolytes in plants include proline, soluble-sugar and protein which play roles in maintaining cellular ionic homeostasis.

On the whole, the calcium signal transduction pathway, phosphatidyl inositol signaling pathway, MAPK signaling pathway, RLKs, and plant hormone signaling pathways play important roles in the response mechanism of *S. pohuashanensis* to high-temperature stress. When *S. pohuashanensis* was subjected to high-temperature stress, the signals were transmitted to cells through transduction pathways, which regulated the expression of TFs and key heat resistance genes. At first, high-temperature stress affected the PM and caused changes in cell membrane fluidity, which activated Ca^2+^ channels and initiated the heat shock signal transduction pathway. At the same time, high temperatures also significantly affected the activity of RLKs on the membrane. Phosphoinositide, as a messenger, induced the release of calcium from the cell, thus rapidly increasing the calcium concentration in the cytoplasm. The signal triggered by high temperatures promoted the combination of Ca^2+^ and CaM, and Ca^2+^-CaM further activated calcium-dependent protein kinase (CDPK) to induce MAPKs to transport signals to the nucleus, and finally induced the expression of HSFs and other heat-resistant genes. cAMP, PLD, and other signaling pathways activated by G protein also played an important role in intercellular signal transmission. IAA and GA might be important plant hormones involved in the regulation of high-temperature morphological formation in *S. pohuashanensis*, and other endogenous hormones such as ABA and SA might also be involved in signal transduction under high-temperature stress, as endogenous signal molecules. However, it was unclear how the signals transport from the MAPK, cAMP, and PLD pathways to plant hormone signaling pathways, and further research would be needed. As the most important regulator, HSFs played an important role in the transcriptional regulatory network of *S. pohuashanensis* in response to high-temperature stress. HsfA3, NAC, ATHB, ERF, and other TFs are the key components that participated in the response of *S. pohuashanensis* to high-temperature stress and played a transcriptional regulatory role by regulating downstream genes. As part of the response to high temperature, HSF transcription factors rapidly induce the expression of Hsps (Hsp70, sHsp) and other cell components dealing with denatured proteins were also induced by high-temperature stress. Then the change of cell osmotic potential leads to the destruction of cell structure, and increase the major of stress-responsive osmolytes in *S. pohuashanensis* including Pro, soluble-sugar and protein, which play roles in maintaining cell morphology and cellular ionic homeostasis. Autophagy may be an intracellular quality control system in *S. pohuashanensis* that removes nonfunctional proteins and damaged cell components. Cell wall modification had positive significance for *S. pohuashanensis* to resist high-temperature. *S. pohuashanensis* activated ROS production upon sensing high-temperature stress. ROS functions might be a signaling molecule for activating local response as well as for transmitting to distal parts. Accumulation of ROS in *S. pohuashanensis* activated HSFs, which in turn activated ROS scavenging and detoxifying enzymes like APX and SOD. However, high-temperature destroyed the ROS homeostasis of *S. pohuanshanensis* and decreased the expression and activity of ROS scavenging enzymes. Detoxification alleviated the toxic state of cells to a certain extent besides. In response to high-temperature stress, *S. pohuashanensis* might enhance its stress resistance through the cooperation of multiple defense mechanisms (Fig. 11).

## Materials and methods

### Plant materials and treatment

One-year-old grafted seedlings from three *S. pohuashanensis* clones were selected from the forest germplasm resources nursery of the National Forest Genetic Resources Platform (NFGR), Beijing University of Agriculture. Three clones were deemed three biological replicates. Each biological replicate contained 10 plants randomly. Seedlings were transferred to a greenhouse under natural light (relative humidity 60%–70%) on March 1st, 2019. Eight weeks later, 2 seedlings from each clones (with 7–8 compound leaves) were selected randomly, then 6 plants were divided into a high-temperature treatment group (HT) and a control group (CK), and placed in an artificial climate chamber (BIC-400; Boxun, Shanghai, China). Seedlings were pretreated with a constant of 16 h/25 °C light, 8 h/18 °C dark, 70% relative humidity, and 180 μmol·m^-2^·s^-1^ light intensity before high-temperature treatment. After 3 d of pretreatment, the HT group was subjected to high-temperature stress at 43 °C for 8 h under light conditions (other conditions unchanged), while the conditions of the CK group remained unchanged. One seedling for each clone was chosen from each group. Chlorophyll fluorescence parameters of HT and CK groups were measured while high temperature treatment at 0, 2, 4, 6 and 8 h. At the end of treatment, all leaves of the HT and CK were collected, frozen in liquid nitrogen, and stored at -80 °C for RNA extraction and physiological measuring.

### Determination of chlorophyll fluorescence parameters

The leaves of HT group and CK group were collected at 0, 2, 4, 6, 8 h after high temperature treatment (the leaves were shaded 20 min before collection). Chlorophyll fluorescence parameters such as the maximum photochemical efficiency (F_v_/F_m_), effective photochemical quantum yield (F_v_'/F_m_'), actual photochemical efficiency of PSII(ΦPSII), photochemical quenching coefficient (qL) and non-photochemical quenching coefficient (NPQ and qN) were measured by PAM-2500 chlorophyll fluorometers (Walz, Nuremberg, Germany).

### Determination of osmoregulation substance content

The leaves of HT group and CK group were collected at 0 and 8 h after high temperature treatment Osmoregulation Substances consisted of MDA, Pro, soluble sugar and soluble total protein in leaves of HT group and CK group were determined by quantitative Kit [Malondialdehyde (MDA) assay kit (TBA method), A003-1–1, Proline assay kit, A107-1–1, Plant soluble sugar content test kit, A45-1–1, The total protein assay kit (BCA method), A045-3–1, Nanjing Jiancheng Bioengineering Institute, Nanjing, China]. The absorbance values of each index were determined by 754 UV–Vis spectrophotometer (automatic) (Shanghai Jinghua, Shanghai, China). The determinations were carried out according to the manufacturer's instructions, and each biological replicate was repeated 5 times.

### Determination of ROS scavenging enzyme activity

Leaves of HT group and CK group were collected at 0 and 8 h after high temperature treatment. Changes of activities of POD, T-SOD, APX, CAT were also detected according to the instruction of matched test kit provided by Nanjing Jiangcheng Bioengineering Institute [Peroxidase assay kit, A084-3–1, Total Superoxide Dismutase (T-SOD) assay kit (Hydroxylamine method), A001-1–1, Ascorbate peroxidase (APX) test kit, A123-1–1, Catalase (CAT) assay kit (Visible light), A007-1–1, Nanjing Jiancheng Bioengineering Institute, Nanjing, China]. The absorbance values of each index were determined by 754 UV–Vis spectrophotometer (automatic). The determinations were carried out according to the manufacturer's instructions, and each biological replicate was repeated 5 times.

### Total RNA isolation, mRNA library construction, sequencing, and transcriptome assembly

RNA samples were extracted from the leaves of *S. pohuashanensis* after 8 h of high temperature stress and control conditions. Total RNA was isolated from the samples using a TransZol Up Plus RNA Kit (Cat ER501-01; Transgen, Beijing, China). The integrity and concentration of total RNA were assessed using an Agilent 2100 Bioanalyzer (Agilent Technologies, Santa Clara, CA, USA) and a Thermo Scientific NanoDrop2000 (Thermo Fisher Scientific, Wilmington, DE, USA). Library preparation and sequencing experiments were performed in accordance with the standard procedure provided by Illumina. Sequencing was performed using an Illumina HiSeq2000 system (Illumina, San Diego, CA, USA) by Shanghai Biotechnology Corporation (Shanghai, China).

Seqtk online software (https://github.com/lh3/seqtk) was used to obtain clean reads after raw reads obtained from sequencing were filtered adaptor sequences in reads, bases with quality Q lower than 30 at the 3 'end, reads with length less than 25, and ribosome RNA reads of the species.. Clean reads from the six libraries were assembled de novo using CLC Genomics Workbench (Version 6.0.4) to produce the primary unigenes ^94,95^. These were then assembled for a second time using CAP3 online stitching software (http://doua.prabi.fr/software/cap3) to acquire the final unigene sequence set and the quality of transcriptome assembly was assessed using BUSCO (Version 3.1.0) based on python^96,97^. These final unigenes were used for further exploration of the transcriptome. The accession number of this project is PRJNA699178.

### Expression profile and enrichment analysis of DEGs

Final unigenes with differential expressions between the HT and CK groups of *S*. *pohuashanensis* were detected using eXpress software (Version 1.5.1) using three replicates per group^98^. Fragments Per Kilobase of Exon Per Million Fragments Mapped (FPKM) was used to calculate the expression abundance of each assembled transcript. Fold-change was calculated according to the FPKM value. The q-value and p-value after correction were obtained using R package edgeR (https://git.bioconductor.org/packages/edgeR^99^). If the Fold-change was much greater than 2 and the q-value was lower than 0.05, the unigene was considered to be differentially expressed.

For annotation, the unigene sequences were searched against the NCBI non-redundant protein database (Nr) using BlastX (http://www.ncbi.nlm.nih.gov/BLAST). Gene Ontology (GO) enrichment analysis of DEGs was performed using Blast2GO (http://www.Blast2GO.com). GO terms with e-values less than 1e-5 were considered significantly enriched in DEGs. We used the Kyoto Encyclopedia of Genes and Genomes (KEGG) KAAS online pathway comparative analysis tools (KAAS, http://www.genome.jp/KEGG/kaas/) to test the statistical enrichment of DEGs in KEGG pathways^100^.

### Validation of DEGs by qRT-PCR

Quantitative RT-PCR was carried out using a Bio-Rad CFX96 real-time PCR detection system (Bio-Rad, Hercules, CA, USA). The specific primers of 20 DEGs were designed using Primer Premier software (version 6.24) and then synthesized by Beijing Ruiboxingke Biotechnology Corporation (Supplementary Table S12). qRT-PCR was carried out using the 2 × T5 Fast qPCR Mix (SYBR Green I) (TSE202; Tsingke, Beijing, China) according to the manufacturer’s protocol. The actin gene of *S. pohuashanensis* was used as an internal marker. A comparative Ct method (2^-△ΔCt^) of relative quantification was used to evaluate the quantitative variation. All quantitative PCRs for each gene preformed three biological replicates with three technical replicates. The RNA samples for quantitative RT-PCR were the same as those used for Illumina sequencing.

### Statistical analysis

Microsoft Excel 2016 (Microsoft Corp., Redmond, WA, USA) and Statistical Product and Service Solutions v.16.0 (SPSS, Chicago, IL, USA) were used to analyze experimental data. Data for P-values were analyzed by Student’s t-test at a significance level of 0.05. The method of Student–Newman–Keuls test (P < 0.05) was used for variance analysis.

### Ethics approval and consent to participate

This study including sample collection was conducted according to China's Biodiversity Conservation Strategy and Action Plan (2011–2030) (Index number: 000014672/2010–00714) and Seed Law of the People's Republic of China (2015 Revised Version), which permits use of biological resources to Chinese for scientific research purpose.

## Supplementary Information

Supplementary Information 1.

Supplementary Information 2.

Supplementary Information 3.

Supplementary Information 4.

Supplementary Information 5.

Supplementary Information 6.
