# Supplementary material for: Physiological and transcriptomic analyses characterized high temperature stress response mechanisms in Sorbus pohuashanensis
Source: Sci Rep. 2021 May 12;11:10117. doi: 10.1038/s41598-021-89418-7 (PMC8115228; doi:10.1038/s41598-021-89418-7)
Supplement: Supplementary file 6 — Supplementary Information 6. [file 41598_2021_89418_MOESM6_ESM.docx]

**Physiological and transcriptomic analyses characterized high temperature stress response mechanisms in *Sorbus* *pohuashanensis***

Xin Pei ^1^, Yan Zhang^1^, Lingyi Zhu ^1^, Dongxue Zhao ^1^, Yizeng Lu ^2^,

Jian Zheng ^13^*

^1^ School of Landscape Architecture, Beijing University of Agriculture, Beijing, 102206, China.

^2^ Shandong Provincial Center of Forest Tree Germplasm Resources, Jinan, Shandong Province, 250102, China.

^3^ Beijing Laboratory of Urban and Rural Ecological Environment, Beijing 100083, China.

^*^ Correspondence: [buazhengjian708@126.com](mailto:buazhengjian708@126.com) (Jian Zheng)


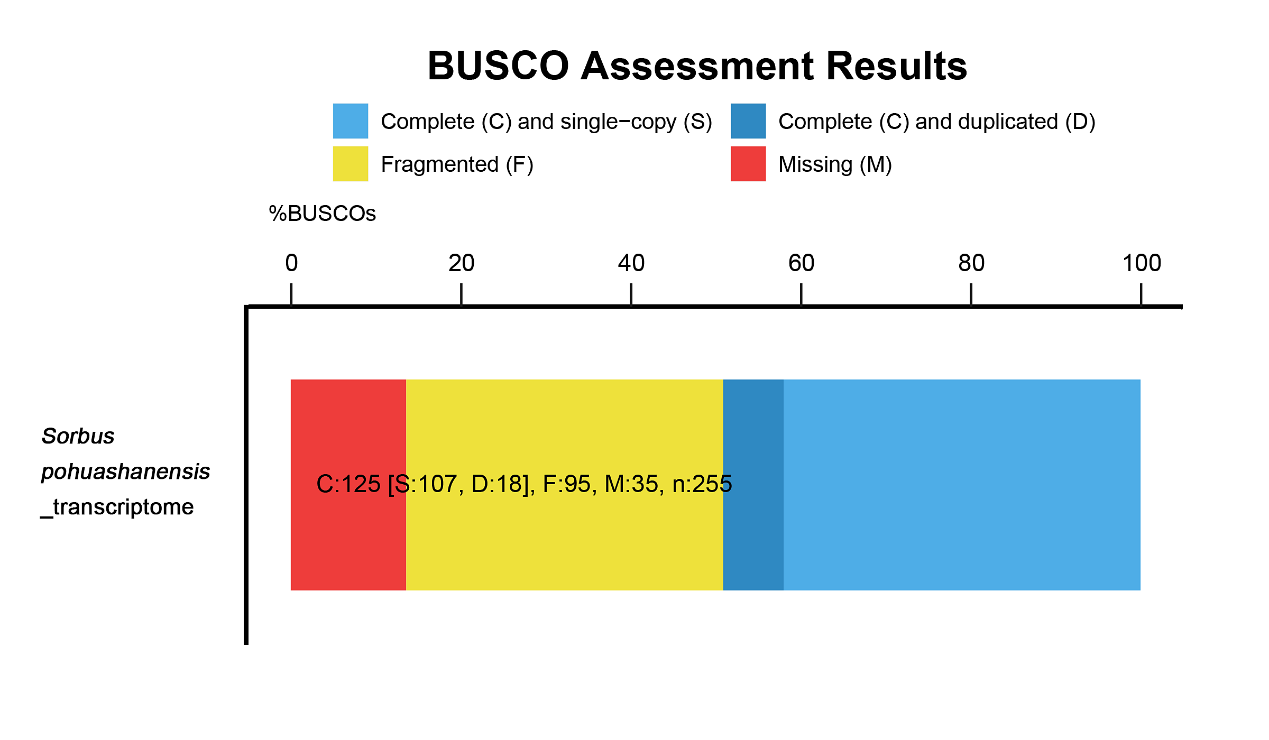
**Figure S1.** BUSCO-based assessment of completeness of the transcriptome assembly of *S. pohuashanensis*.

The lineage dataset is: eukaryota_odb10 (Creation date: 2020-09-10, number of species: 70, number of BUSCOs: 255).

**
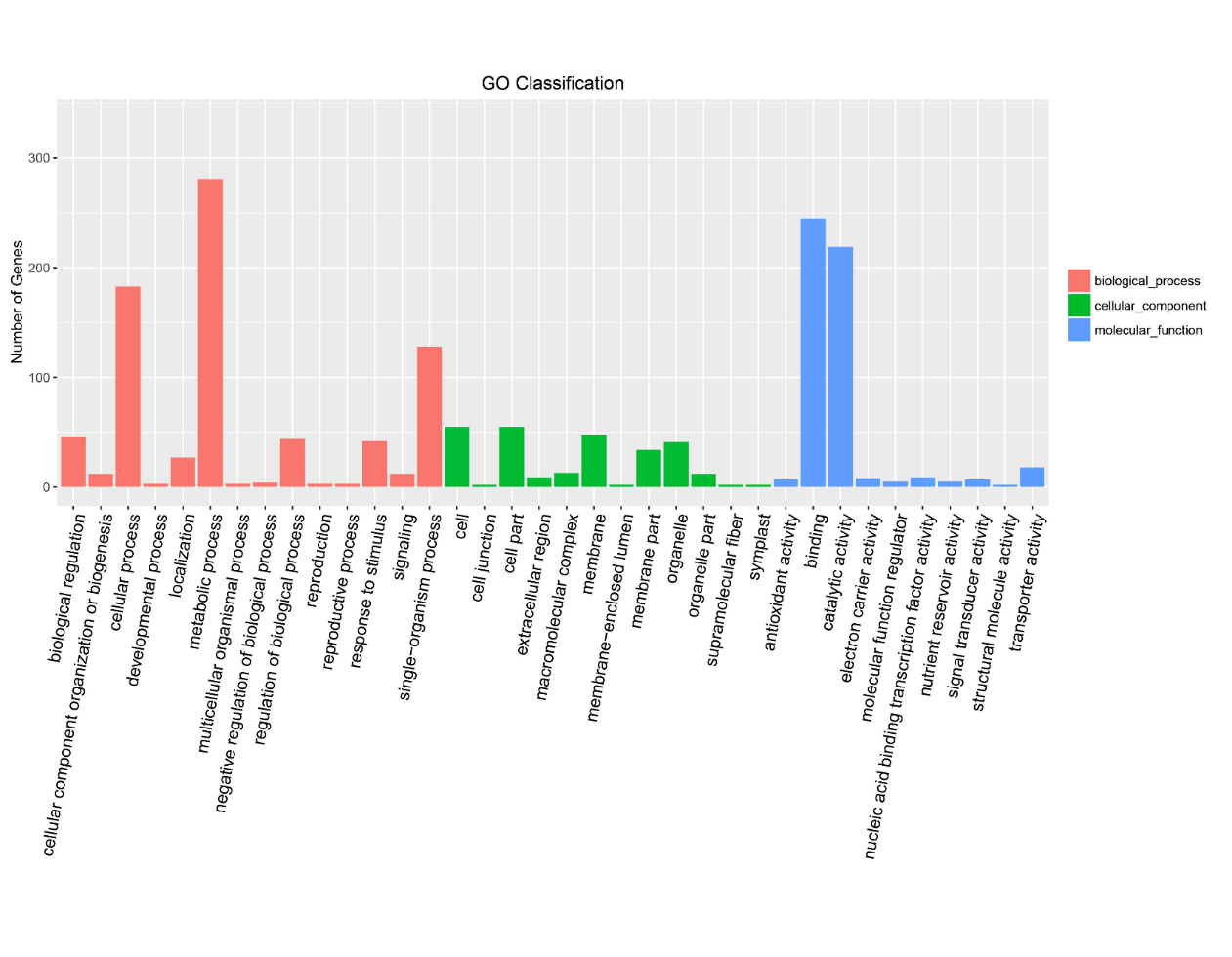
Supplementary** **material**

**Figure S2.** GO classification of differential expression genes under high-temperature treatment. GO enrichment analysis was performed using the agriGO analysis tool. Only GO terms with significant levels of enrichment (q-value≤5%) are shown.

| Table S1. resumes of raw RNA-seq data. | | | | | | |
| --- | --- | --- | --- | --- | --- | --- |
| Sample | Raw Reads(Million) | Clean Reads(Million) | Valid Bases (%) | Q30 (%) | GC (%) | rRNA ratio(%) |
| CK1 | 55.82 | 53.95 | 96.65 | 94.33 | 46.00 | 11.64 |
| CK2 | 56.11 | 54.17 | 96.54 | 94.20 | 46.00 | 1.42 |
| CK3 | 47.71 | 46.15 | 96.73 | 94.07 | 46.00 | 1.20 |
| HT1 | 47.88 | 45.99 | 96.04 | 94.12 | 46.00 | 17.85 |
| HT2 | 56.30 | 54.33 | 96.49 | 94.37 | 46.00 | 8.10 |
| HT3 | 56.94 | 54.76 | 96.18 | 94.48 | 46.00 | 21.65 |
| means | 53.46 | 51.56 | 96.44 | 94.26 | 46.00 | 10.31 |

| Table S2. Summary of assembly statistics for *S. pohuashanensis* leaf transcriptome. | | | | | | | |
| --- | --- | --- | --- | --- | --- | --- | --- |
| Statistics | counts | Total length (bp) | N50 (bp) | Average length (bp) | longest (bp) | N(%) | GC(%) |
| contigs | 229,107 | 103,394,470 | 516 | 451 | 11,105 | 1.5 | 40.9 |
| Primary Unigenes | 197,028 | 104,997,728 | 631 | 533 | 14,880 | 1.5 | 40.9 |
| Final Unigenes | 130,003 | 85,643,039 | 838 | 658.78 | 14,880 | 1.76 | 40.82 |

Table.S3. Expression of unigene under high-temperature stress.

Please view this table in an individual file.

Table S4. Differential expression genes of unigene under high-temperature stress.

Please view this table in an individual file.

Table S5. Unigene annotated by Non-Redundant Protein Sequence Database.

Please view this table in an individual file.

Table S6. GO annotation of DEGs under high-temperature stress.

Please view this table in an individual file.

Table S7. KEGG annotation of DEGs under high-temperature stress.

Please view this table in an individual file.

| Table S8.DEGs related to Signal transduction pathway under high-temperature stress | | | |
| --- | --- | --- | --- |
| Gene | Unigene id | Description | UP/DOWN |
| *ACA13* | Contig_29625 | calcium-transporting ATPase 13, plasma membrane-type | UP |
|  | Contig_27953 | calcium-transporting ATPase 13, plasma membrane-type | UP |
| *CBL10* | Contig_15961 | calcineurin B-like protein 10 | UP |
| *CCAMK-like* | First_Contig19732 | calcium/calmodulin-dependent serine/threonine-protein kinase-like | UP |
| *MCUb-like* | Contig_102271 | mitochondrial calcium uniporter regulatory subunit MCUb-like | UP |
|  | Contig_102272 | mitochondrial calcium uniporter regulatory subunit MCUb-like | UP |
| *MICU1-like* | Second_Contig3253 | calcium uptake protein 1, mitochondrial-like | UP |
| *PLC2-like* | Contig_9830 | phosphoinositide phospholipase C 2-like | UP |
|  | Contig_15993 | phosphoinositide phospholipase C 2-like | DOWN |
| *CPK26-like* | First_Contig1922 | calcium-dependent protein kinase 26-like | DOWN |
| *CML50* | Contig_145120 | calcium-binding protein CML50, partial | DOWN |
| *CRT3-like* | Second_Contig781 | calreticulin-3-like | DOWN |
|  | Second_Contig913 | calreticulin-3-like | DOWN |
| *PBP1-like* | First_Contig11642 | calcium-binding protein PBP1-like | DOWN |
| *ARF* | First_Contig227 | ADP-ribosylation factor | DOWN |
| *BIG5-like* | Second_Contig1259 | brefeldin A-inhibited guanine nucleotide-exchange protein 5-like | DOWN |
| *FTSZ1-like* | Contig_37734 | cell division protein FtsZ homolog 1, chloroplastic-like | DOWN |
| *NOA1* | Contig_48435 | NO-associated protein 1, chloroplastic/mitochondrial | DOWN |
| *SAR1A* | Contig_18924 | GTP-binding protein SAR1A | DOWN |
| *YDA-like* | Contig_50904 | mitogen-activated protein kinase kinase kinase YODA-like | UP |
| *UBC28-like* | Contig_58426 | ubiquitin-conjugating enzyme E2 28-like | DOWN |
|  | Second_Contig3172 | ubiquitin-conjugating enzyme E2 28-like | DOWN |
| *PLDδ-like* | First_Contig303 | phospholipase D delta-like | UP |
|  | Contig_54710 | phospholipase D delta-like | UP |
| *ACX1-like* | Contig_32104 | peroxisomal acyl-coenzyme A oxidase 1-like | DOWN |
|  | First_Contig1891 | peroxisomal acyl-coenzyme A oxidase 1-like | DOWN |
| *NIK1* | Contig_24250 | LRR receptor-like serine/threonine-protein kinase At1g06840 | UP |
|  | Contig_33555 | LRR receptor-like serine/threonine-protein kinase At1g06840 | UP |
|  | First_Contig16921 | LRR receptor-like serine/threonine-protein kinase At1g06840 | UP |
| *CEPR1-like* | Contig_26908 | LRR receptor-like serine/threonine-protein kinase At1g53420 | DOWN |
|  | Contig_64320 | LRR receptor-like serine/threonine-protein kinase At1g53420 | DOWN |
| *CLV1-like* | First_Contig17706 | receptor protein kinase CLAVATA1-like | DOWN |
| LOC103400642*-like* | Contig_5568 | inactive leucine-rich repeat receptor-like protein kinase At3g28040 | DOWN |
| LOC103427118*-like* | Contig_26162 | LRR receptor-like serine/threonine-protein kinase At1g56140 | DOWN |
| LOC103432629*-like* | Contig_124173 | LRR receptor-like serine/threonine-protein kinase At3g47570 | UP |
|  | Contig_143475 | LRR receptor-like serine/threonine-protein kinase At3g47570 | UP |
| LOC114827494*-like* | Contig_8294 | LRR receptor-like serine/threonine-protein kinase At1g53440 | DOWN |
|  | Contig_26967 | LRR receptor-like serine/threonine-protein kinase At1g53440 | DOWN |
|  | First_Contig5366 | LRR receptor-like serine/threonine-protein kinase At1g53440 | DOWN |
| *Lr10-like* | Contig_12744 | G-type lectin S-receptor-like serine/threonine-protein kinase At1g34300 | DOWN |
| *LRR-RLK* | Contig_7642 | leucine-rich repeat receptor-like protein kinase At1g35710 | DOWN |
| *RLK7* | First_Contig4766 | LRR receptor-like serine/threonine-protein kinase At1g07650 | DOWN |
| *RLP12* | Contig_61891 | receptor-like protein 12 | DOWN |
| *UVR8-like* | First_Contig12454 | ultraviolet-B receptor UVR8-like | UP |
| *ABP19a* | Contig_1211 | auxin-binding protein ABP19a | DOWN |
|  | Contig_1365 | auxin-binding protein ABP19a | DOWN |
| *IAA6* | Contig_107942 | auxin-induced protein IAA6 | DOWN |
| *GA2-like* | First_Contig18218 | ent-kaur-16-ene synthase, chloroplastic-like isoform X6 | UP |
| *GAI-like* | Contig_17860 | DELLA protein GAI-like | DOWN |
| *GASA1-like* | Contig_20194 | gibberellin-regulated protein 1-like | DOWN |
| *NCED1-like* | First_Contig13764 | 9-cis-epoxycarotenoid dioxygenase NCED1, chloroplastic-like | UP |
| *NCED3-like* | Contig_81000 | 9-cis-epoxycarotenoid dioxygenase NCED3, chloroplastic-like | UP |
| *SAMT-like* | Contig_21633 | salicylate carboxymethyltransferase-like | DOWN |
|  | First_Contig9252 | salicylate carboxymethyltransferase-like | DOWN |
| *PED1* | Contig_31873 | 3-ketoacyl-CoA thiolase 2, peroxisomal | DOWN |
| *BSK2-like* | Contig_31943 | serine/threonine-protein kinase BSK2-like | DOWN |

| Table S9.DEGs related to Transcriptional regulation in under high-temperature stress | | | |
| --- | --- | --- | --- |
| Gene | Unigene id | Description | UP/DOWN |
| *NAC2* | Contig_39015 | NAC domain-containing protein 2 | UP |
|  | Contig_74758 | NAC domain-containing protein 2 | UP |
| *NAC2-like* | First_Contig8679 | NAC domain-containing protein 2-like | UP |
|  | First_Contig9153 | NAC domain-containing protein 2-like | UP |
| *NAC12-like* | Contig_56091 | NAC domain-containing protein 12-like isoform X3 | DOWN |
|  | First_Contig2838 | NAC domain-containing protein 12-like isoform X3 | DOWN |
| *NAC29* | Contig_133259 | NAC transcription factor 29 | UP |
| *NAC29-like* | First_Contig15218 | NAC transcription factor 29-like | UP |
| *NAC68-like* | Contig_85800 | NAC domain-containing protein 68-like | UP |
| *NAC72* | First_Contig21896 | NAC domain-containing protein 72, partial | UP |
| *HsfA3-like* | First_Contig4704 | heat stress transcription factor A-3-like | UP |
|  | First_Contig8678 | heat stress transcription factor A-3-like | UP |
| *Hsp70-like* | First_Contig7750 | heat shock 70 kDa protein-like | UP |
| *Hsp70-17-like* | Contig_4643 | heat shock 70 kDa protein 17-like | DOWN |
|  | Second_Contig54 | heat shock 70 kDa protein 17-like | DOWN |
|  | Second_Contig1545 | heat shock 70 kDa protein 17-like | DOWN |
| *sHsp-like* | Contig_63802 | small heat shock protein, chloroplastic-like | UP |
| *NF-YB3* | Contig_38433 | nuclear transcription factor Y subunit B-3-like | UP |
|  | Contig_60233 | nuclear transcription factor Y subunit B-3-like | UP |
| LOC103964362*-like* | First_Contig5393 | F-box/kelch-repeat protein At1g15670-like | UP |
| LOC103409567*-like* | Contig_54048 | F-box/kelch-repeat protein At2g44130-like | UP |
|  | First_Contig8329 | F-box/kelch-repeat protein At2g44130-like | UP |
|  | First_Contig8335 | F-box/kelch-repeat protein At2g44130-like | UP |
|  | First_Contig8802 | F-box/kelch-repeat protein At2g44130-like | UP |
|  | First_Contig14994 | F-box/kelch-repeat protein At2g44130-like | UP |
| LOC103451255*-like* | Contig_20606 | F-box/kelch-repeat protein At3g06240-like | UP |
| *FBL14-like* | Contig_41230 | F-box/LRR-repeat protein 14-like | UP |
|  | First_Contig6491 | F-box/LRR-repeat protein 14-like | UP |
| *MAX2A* | Contig_70793 | F-box/LRR-repeat MAX2 homolog A | UP |
|  | Contig_79728 | F-box/LRR-repeat MAX2 homolog A-like | UP |
| *SKIP23-like* | First_Contig25018 | F-box protein SKIP23-like | UP |
| *ATHB6-like* | Contig_39631 | homeobox-leucine zipper protein ATHB-6-like | DOWN |
|  | First_Contig6201 | homeobox-leucine zipper protein ATHB-6-like | DOWN |
| *ATHB12-like* | Second_Contig3380 | homeobox-leucine zipper protein ATHB-12-like | UP |
| *ERF011-like* | First_Contig14666 | ethylene-responsive transcription factor ERF011-like | UP |
| *ERF113* | Contig_1148 | ethylene-responsive transcription factor ERF113 | DOWN |
| *BIP5* | Contig_430 | luminal-binding protein 5 | DOWN |
|  | Contig_5803 | luminal-binding protein 5 | DOWN |
|  | Second_Contig1675 | luminal-binding protein 5 | DOWN |
| *BIP5-like* | Contig_78 | luminal-binding protein 5-like | DOWN |
|  | Contig_2520 | luminal-binding protein 5-like | DOWN |
|  | Second_Contig901 | luminal-binding protein 5-like | DOWN |
| *b-ZIP60* | Contig_404 | bZIP transcription factor 60 | DOWN |
|  | Contig_405 | bZIP transcription factor 60 | DOWN |
|  | First_Contig52 | bZIP transcription factor 60 | DOWN |
| *GL2-like* | Contig_19434 | homeobox-leucine zipper protein GLABRA 2-like | DOWN |

| Table S10.DEGs related to protein and ROS homeostasis under high-temperature stress. | | | |
| --- | --- | --- | --- |
| Gene | Unigene id | Description | UP/DOWN |
| *GAUT3* | First_Contig3351 | probable galacturonosyltransferase 3 | UP |
|  | Contig_36564 | probable galacturonosyltransferase 3 | UP |
| *GAUT12* | Contig_128070 | probable galacturonosyltransferase 12 | UP |
| *CSLC5* | Contig_27207 | probable xyloglucan glycosyltransferase 5 | UP |
| *GRPA3-like* | First_Contig15226 | glycine-rich protein A3-like | UP |
| *TBL1* | Contig_72047 | protein trichome birefringence-like 1 | DOWN |
| *TBL19* | First_Contig8881 | protein trichome birefringence-like 19 | DOWN |
| *TBL38* | Contig_4698 | protein trichome birefringence-like 38 | DOWN |
|  | Second_Contig133 | protein trichome birefringence-like 39 | DOWN |
| *TBL6* | First_Contig15582 | protein trichome birefringence-like 6 | DOWN |
| *XTH23* | Contig_38035 | probable xyloglucan endotransglucosylase/hydrolase protein 23 | DOWN |
| *GRP-like* | Contig_6712 | glycine-rich cell wall structural protein-like | DOWN |
| *HHT1-like* | First_Contig26102 | omega-hydroxypalmitate O-feruloyl transferase-like | UP |
| *CER26-like* | Contig_36763 | protein ECERIFERUM 26-like | DOWN |
| *PME35* | Contig_54034 | probable pectinesterase/pectinesterase inhibitor 35 | DOWN |
| *PME-like* | Contig_4487 | pectinesterase-like | DOWN |
|  | Contig_4488 | pectinesterase-like | DOWN |
| *GLR2.2* | Contig_100376 | glutamate receptor 2.2-like | UP |
| *GLR2.8* | Contig_3584 | glutamate receptor 2.8-like | UP |
|  | Contig_163288 | glutamate receptor 2.8-like | UP |
|  | Contig_2806 | glutamate receptor 2.8-like | UP |
| *abcG11-like* | Contig_37118 | ABC transporter G family member 11-like | DOWN |
| *abcG22-like* | First_Contig3281 | ABC transporter G family member 22-like | DOWN |
|  | Contig_17645 | ABC transporter G family member 22-like | DOWN |
| *COPB1-like* | Second_Contig832 | coatomer subunit beta-1-like | DOWN |
|  | First_Contig28 | coatomer subunit beta-1-like | DOWN |
|  | Contig_7338 | coatomer subunit beta-1-like | DOWN |
|  | Contig_7339 | coatomer subunit beta-1-like | DOWN |
|  | Contig_2523 | coatomer subunit beta-1-like | DOWN |
| *GSNAP* | Contig_41936 | gamma-soluble NSF attachment protein | DOWN |
| *VSR6-like* | First_Contig4020 | vacuolar-sorting receptor 6-like | DOWN |
|  | Contig_14207 | vacuolar-sorting receptor 6-like | DOWN |
| *NPF3.1* | First_Contig529 | protein NRT1/ PTR FAMILY 3.1 | DOWN |
| *NPF3.1-like* | Second_Contig895 | protein NRT1/ PTR FAMILY 3.1-like | DOWN |
| *NPF5.1* | Contig_47214 | protein NRT1/ PTR FAMILY 5.1 | DOWN |
| *NPF5.6-like* | Contig_4089 | protein NRT1/ PTR FAMILY 5.6-like | DOWN |
|  | Contig_16551 | protein NRT1/ PTR FAMILY 5.6-like | DOWN |
| *TIP1-3* | First_Contig11919 | aquaporin TIP1-3 | DOWN |
| *TIP1-3-like* | First_Contig20477 | aquaporin TIP1-3-like | DOWN |
| *TT12* | Second_Contig291 | protein TRANSPARENT TESTA 12 | DOWN |
| *TT12-like* | Contig_16714 | protein TRANSPARENT TESTA 12-like | DOWN |
|  | Contig_77208 | protein TRANSPARENT TESTA 12-like | DOWN |
| *VIII-1* | Contig_189257 | myosin-1-like isoform X2 | UP |
| *VIII-B-like* | First_Contig261 | myosin-4-like | DOWN |
|  | Contig_1963 | myosin-4-like | DOWN |
| *XI-E-like* | Contig_53580 | myosin-11-like | UP |
| *ATJ11-like* | Contig_53431 | chaperone protein dnaJ 11, chloroplastic-like | UP |
|  | First_Contig8694 | chaperone protein dnaJ 11, chloroplastic-like | UP |
|  | First_Contig900 | chaperone protein dnaJ 11, chloroplastic-like | UP |
|  | Contig_55881 | chaperone protein dnaJ 11, chloroplastic-like | UP |
|  | First_Contig26228 | chaperone protein dnaJ 11, chloroplastic-like | UP |
|  | First_Contig10340 | chaperone protein dnaJ 11, chloroplastic-like | UP |
| *ATJ6-like* | Contig_97111 | chaperone protein dnaJ 6-like | UP |
| *BAG2-like* | Contig_11444 | BAG family molecular chaperone regulator 2-like | UP |
| *GI9-like* | Contig_267 | glucan endo-1,3-beta-glucosidase, acidic isoform GI9-like | UP |
| *RGA2-like* | Contig_2426 | disease resistance protein RGA2-like | DOWN |
|  | Second_Contig1052 | disease resistance protein RGA2-like | DOWN |
|  | Contig_3126 | disease resistance protein RGA2-like | DOWN |
|  | Contig_37978 | disease resistance protein RGA2-like | DOWN |
|  | Contig_2459 | disease resistance protein RGA2-like | DOWN |
|  | Contig_33046 | disease resistance protein RGA2-like | DOWN |
| *RGA2-like* | Contig_35130 | disease resistance protein RGA2-like | UP |
|  | First_Contig23125 | disease resistance protein RGA2-like | UP |
|  | Contig_58195 | disease resistance protein RGA2-like | UP |
| *RGA3* | Contig_81 | putative disease resistance protein RGA3 | UP |
| *AMP2-2* | First_Contig2649 | vicilin-like antimicrobial peptides 2-2 | UP |
| *TDX-like* | First_Contig118 | TPR repeat-containing thioredoxin TDX-like isoform X2 | DOWN |
|  | Contig_26212 | TPR repeat-containing thioredoxin TDX-like isoform X2 | DOWN |
|  | Contig_884 | TPR repeat-containing thioredoxin TDX-like isoform X2 | DOWN |
| *FKBP53-like* | Contig_3338 | peptidyl-prolyl cis-trans isomerase FKBP53-like | DOWN |
| *ATG8C-like* | Contig_62877 | autophagy-related protein 8C-like | DOWN |
| *uspA-like* | Second_Contig854 | universal stress protein A-like protein | DOWN |
| *CYP714C2-like* | First_Contig16716 | cytochrome P450 714C2-like | DOWN |
| *CYP71A1-like* | Contig_35251 | cytochrome P450 71A1-like | UP |
| *CYP71A1-like* | First_Contig10170 | cytochrome P450 71A1-like | DOWN |
|  | Contig_66445 | cytochrome P450 71A2-like | DOWN |
| *CYP71AV8-like* | First_Contig2576 | cytochrome P450 71AV8-like | UP |
|  | Contig_24347 | cytochrome P450 71AV8-like | UP |
|  | Contig_8341 | cytochrome P450 71AV8-like | UP |
|  | Contig_38518 | cytochrome P450 71AV8-like | UP |
|  | Contig_6565 | cytochrome P450 71AV8-like | UP |
|  | Contig_8969 | cytochrome P450 71AV8-like | UP |
|  | Contig_33432 | cytochrome P450 71AV8-like | UP |
|  | Contig_27776 | cytochrome P450 71AV8-like | UP |
|  | First_Contig25137 | cytochrome P450 71AV8-like | UP |
|  | First_Contig25316 | cytochrome P450 71AV8-like | UP |
|  | Contig_24348 | cytochrome P450 71AV8-like | UP |
|  | First_Contig19768 | cytochrome P450 71AV8-like | UP |
| *CYP72A219-like* | Contig_65461 | cytochrome P450 CYP72A219-like | DOWN |
| *CYP749A22-like* | Contig_45515 | cytochrome P450 CYP749A22-like | UP |
|  | Contig_64703 | cytochrome P450 CYP749A22-like | UP |
| *CYP77A1-like* | Contig_167267 | cytochrome P450 77A1-like | UP |
| *CYP79A1-like* | Contig_6715 | tyrosine N-monooxygenase-like, partial | DOWN |
| *CYP79D3* | Contig_6714 | isoleucine N-monooxygenase 1-like | DOWN |
|  | Contig_76030 | isoleucine N-monooxygenase 1-like | DOWN |
| *CYP81D11-like* | Contig_90146 | cytochrome P450 81D11-like | UP |
| *CYP82A3-like* | First_Contig10826 | cytochrome P450 82A3-like | DOWN |
| *CYP82G1-like* | First_Contig4049 | cytochrome P450 82G1-like isoform X2 | DOWN |
| *CYP85A* | First_Contig13451 | cytochrome P450 85A | DOWN |
| *CYP90A1* | Contig_5703 | cytochrome P450 90A1 | DOWN |
| *CYP90A1-like* | Contig_5702 | cytochrome P450 90A1-like isoform X2 | DOWN |
| *CYP94C1-like* | First_Contig16148 | cytochrome P450 94C1-like | DOWN |
| *AKR4C9-like* | Contig_2433 | aldo-keto reductase family 4 member C9-like | UP |
| *idnD-like* | Contig_44287 | L-idonate 5-dehydrogenase-like | UP |
| *AAO-like* | Contig_54363 | L-ascorbate oxidase-like | DOWN |
|  | Contig_8569 | L-ascorbate oxidase-like | DOWN |
| *GSTU17-like* | Contig_16520 | glutathione S-transferase U17-like | DOWN |
| *GSTU9-like* | First_Contig3634 | glutathione S-transferase U9-like | UP |
| *MDAR5* | Second_Contig1652 | monodehydroascorbate reductase, chloroplastic isoform X2 | DOWN |
|  | Contig_3416 | monodehydroascorbate reductase, chloroplastic isoform X2 | DOWN |
|  | Contig_11677 | monodehydroascorbate reductase, chloroplastic isoform X2 | DOWN |
|  | Contig_19698 | monodehydroascorbate reductase, chloroplastic isoform X2 | DOWN |
| *PER47* | Second_Contig1004 | peroxidase 47 | DOWN |
| *PER47-like* | Contig_69694 | peroxidase 47-like | DOWN |
| *PERP7-like* | First_Contig3211 | peroxidase P7-like | DOWN |
|  | Contig_101526 | peroxidase P7-like | DOWN |
|  | Contig_101525 | peroxidase P7-like | DOWN |
| *PNC1-like* | First_Contig9974 | cationic peroxidase 1-like | DOWN |
|  | First_Contig9970 | cationic peroxidase 1-like | DOWN |
| *AOX3-like* | Contig_7053 | alternative oxidase 3, mitochondrial-like | DOWN |
| *PPO-like* | Contig_7042 | polyphenol oxidase, chloroplastic-like | UP |
| *PQOR* | First_Contig11418 | putative quinone-oxidoreductase homolog, chloroplastic | DOWN |

| Table S11. DEGs related to metabolic pathway under high-temperature stress | | |  |  |
| --- | --- | --- | --- | --- |
| Gene | Unigene id | Description | | UP/DOWN |
| *CA2* | Contig_21574 | carbonic anhydrase 2 isoform X2 | | DOWN |
|  | Contig_14123 | carbonic anhydrase 2 isoform X2 | | DOWN |
| *CA2-like* | Contig_18850 | carbonic anhydrase 2-like isoform X1 | | DOWN |
| *AMY2* | Second_Contig2310 | probable alpha-amylase 2 | | DOWN |
| *BAM1* | Contig_22471 | beta-amylase 1, chloroplastic | | UP |
| *BAM1-like* | First_Contig3322 | beta-amylase 1, chloroplastic-like | | UP |
| *BGAL* | Contig_30707 | beta-galactosidase | | UP |
|  | Contig_1214 | beta-galactosidase | | UP |
|  | First_Contig5388 | beta-galactosidase | | UP |
|  | Contig_32721 | beta-galactosidase | | UP |
| *GAPB* | Contig_7871 | glyceraldehyde-3-phosphate dehydrogenase B, chloroplastic-like isoform X3 | | UP |
| *GBSS1-like* | Contig_118270 | granule-bound starch synthase 1, chloroplastic/amyloplastic-like | | UP |
| *GBSS1-like* | First_Contig16319 | granule-bound starch synthase 1, chloroplastic/amyloplastic-like | | DOWN |
| *GWD2* | Contig_3957 | alpha-glucan water dikinase 2 isoform X2 | | DOWN |
| *LSF2* | Contig_17850 | phosphoglucan phosphatase LSF2, chloroplastic | | DOWN |
| *PP2A4-like* | Contig_5735 | uncharacterized protein PHLOEM PROTEIN 2-LIKE A4-like | | DOWN |
| *PP2A9-like* | Contig_3685 | protein PHLOEM PROTEIN 2-LIKE A9-like | | DOWN |
| *GML* | Contig_33193 | aldose 1-epimerase | | DOWN |
| *UGE1* | Contig_2658 | bifunctional UDP-glucose 4-epimerase and UDP-xylose 4-epimerase 1 | | UP |
|  | First_Contig12634 | bifunctional UDP-glucose 4-epimerase and UDP-xylose 4-epimerase 1 | | UP |
| *UGE1-like* | Contig_2657 | bifunctional UDP-glucose 4-epimerase and UDP-xylose 4-epimerase 1-like | | UP |
| *UGT85A2-like* | Contig_6821 | UDP-glycosyltransferase 85A2-like | | DOWN |
|  | First_Contig1255 | UDP-glycosyltransferase 85A2-like | | DOWN |
|  | Second_Contig59 | UDP-glycosyltransferase 85A2-like | | DOWN |
|  | Contig_5337 | UDP-glycosyltransferase 85A2-like | | DOWN |
| *UGT87A1-like* | First_Contig11524 | UDP-glycosyltransferase 87A1-like | | DOWN |
|  | Contig_2100 | UDP-glycosyltransferase 87A1-like | | DOWN |
| *PDCB4* | Contig_41842 | glucan endo-1,3-beta-glucosidase-like protein At1g69295 | | DOWN |
| *SDRB-like* | Contig_36738 | peroxisomal 2,4-dienoyl-CoA reductase-like | | UP |
| *GDSL-like* | Contig_32783 | GDSL esterase/lipase At3g26430-like | | DOWN |
| *PES2-like* | First_Contig7664 | acyltransferase-like protein At3g26840, chloroplastic | | DOWN |
|  | Contig_49229 | acyltransferase-like protein At3g26840, chloroplastic | | DOWN |
| *CXE15* | Contig_38193 | probable carboxylesterase 15 | | DOWN |
|  | Contig_80307 | probable carboxylesterase 15 | | DOWN |
| *GDE1-like* | First_Contig10249 | glycerophosphodiester phosphodiesterase GDE1-like | | UP |
|  | Contig_34421 | glycerophosphodiester phosphodiesterase GDE1-like | | UP |
| *GPP2* | Second_Contig272 | (DL)-glycerol-3-phosphatase 2 | | DOWN |
| *LACS2* | Second_Contig62 | long chain acyl-CoA synthetase 2 | | DOWN |
| *LACS4* | Contig_18455 | long chain acyl-CoA synthetase 4 | | DOWN |
| *PXG4* | First_Contig2929 | probable peroxygenase 4 | | DOWN |
| *NIA* | Contig_1484 | nitrate reductase [NADH] | | DOWN |
|  | First_Contig10116 | nitrate reductase [NADH] | | DOWN |
| *NIA-like* | Contig_23177 | nitrate reductase [NADH]-like | | DOWN |
|  | First_Contig1775 | nitrate reductase [NADH]-like | | DOWN |
| *PYD2-like* | First_Contig16391 | dihydropyrimidinase-like | | UP |
| *ARD1-like* | Contig_12154 | 1,2-dihydroxy-3-keto-5-methylthiopentene dioxygenase 1-like | | UP |
| *ASN-like* | Contig_46643 | asparagine synthetase [glutamine-hydrolyzing]-like | | UP |
| *CLPP-like* | First_Contig4937 | 3-hydroxyisobutyryl-CoA hydrolase-like protein 5 | | DOWN |
| *GDH2* | First_Contig21191 | glutamate dehydrogenase 2 | | UP |
| *TAT2* | Contig_25220 | probable aminotransferase TAT2 | | DOWN |
| *ERVB-like* | Contig_190023 | ervatamin-B-like | | UP |
| *PLR1* | First_Contig13279 | pyridoxal reductase, chloroplastic isoform X1 | | UP |
| *PMAT2-like* | Contig_4770 | phenolic glucoside malonyltransferase 2-like | | DOWN |
| *QPT* | First_Contig4326 | nicotinate-nucleotide pyrophosphorylase [carboxylating], chloroplastic | | DOWN |
| *CPC-like* | Second_Contig1597 | cucumber peeling cupredoxin-like | | DOWN |
| *ENODL2* | Contig_4978 | early nodulin-like protein 2 isoform X1 | | DOWN |
| *cob* | Contig_190182 | apocytochrome b (mitochondrion) | | UP |
| *cox1* | Contig_190040 | cytochrome c oxidase subunit 1 (mitochondrion) | | UP |
| *cox2* | Contig_190147 | cytochrome c oxidase subunit 2 (mitochondrion) | | UP |
| *cox3* | Contig_190111 | cytochrome c oxidase subunit 3 (mitochondrion) | | UP |
| *LFNR-like* | Contig_7298 | ferredoxin--NADP reductase, leaf isozyme, chloroplastic-like | | DOWN |
| *BCS1-A* | Contig_182529 | probable mitochondrial chaperone BCS1-A | | UP |
| *BCS1-like* | Second_Contig1287 | mitochondrial chaperone BCS1-like | | DOWN |
| *FTSH4* | First_Contig7978 | ATP-dependent zinc metalloprotease FTSH 4, mitochondrial | | DOWN |
|  | First_Contig136 | ATP-dependent zinc metalloprotease FTSH 4, mitochondrial | | DOWN |
| *MPC2-like* | Contig_19330 | mitochondrial pyruvate carrier 2-like | | DOWN |
| *NADP-ME* | Contig_31834 | NADP-dependent malic enzyme isoform X2 | | DOWN |
|  | Contig_1434 | NADP-dependent malic enzyme isoform X2 | | DOWN |
| *PSBR-like* | Contig_14744 | photosystem II 10 kDa polypeptide, chloroplastic-like | | UP |
| *TKL* | Contig_3003 | transketolase, chloroplastic | | DOWN |
| *ABCG-like* | First_Contig23273 | pleiotropic drug resistance protein 1-like isoform X1 | | DOWN |
| *ATP6* | Contig_190132 | ATP synthase F0 subunit 6 (mitochondrion) | | UP |
| *atpB* | First_Contig366 | ATP synthase CF1 beta subunit | | UP |
| *ATR3-like* | Contig_111685 | NADPH-dependent diflavin oxidoreductase 1-like | | UP |

| Table S12. List of primers used in the quantitative RT-PCR | | |  |
| --- | --- | --- | --- |
| Genes | | Forward Primer（5’-3’） | Reverse Primer（5’-3’） |
| *ActinQ* | | TGGATGGCTGGAAGAGGA | GAGCGGGAAATTGTGAGG |
| *bHLH66-like* | | GCAACAACAACCAAGTCATCC | AGTAAATCCAAAGCCACAAGGT |
| *ERF011-like* | | CCGTCGTCTTGGAATACA | AAGCAATACAGAGGAATACG |
| *ERF113* | | CAAGTAGTAGTGGTGCTTCCTC | GGCTGCTGCGACATCATTA |
| *Hsp70-like* | | GAAGAAGTGGTTGGACGAGAG | GGTTTCATTGCTTGTGGGTTT |
| *HsfA3-like* | | GCAGCATGAACTCGGTAA | TGTCGTAACTTGTGAAGGAT |
| *NAC29* | | TCTGGAAGGCGGTATTCA | TTGGAAGGCAACTGGAAC |
| *NAC72* | | AAGAGCAAGATGACGATGAG | AAGGCACCAAGACCAACT |
| *ABP19a* | | TGACCTTAGCAGGAATCTTG | ACACAATCACTCATCACTTC |
| *CBL10* | | GAAGCGATGGATGCCGAAG | TTGAGCGAGAAGAGGTTAAGC |
| *GA2* | | ATTATTGACGGCTTCGCTCTT | CAGTGGACGGATGTTTGGG |
| *GASA1-like* | | GGGTTAAACGCAATTTCTGT | CCTTGAATCAATCCTCTGTCT |
| *NCED1-like* | | AGAGAATCTGAACCTGGAAG | GCCATAGATATGCTTCTTCAC |
| *PBP1-like* | | CAGGACTCAGTCTGAACAT | GGTCATTACGCTGGAGAG |
| *BIP5(Hsp70)* | | TCCAAGCCAGCATCATCCATA | AGCACTCAGTAGCCAGCAC |
| *CYP71AV8-like* | | GCAGCGACCTCTTCTCCTAA | AGCCAAATCCCAAATACACCAA |
| *GSTU9-like* | | TCTTCACGGCGGAGATTCT | CTGCTTGGTAAACTGGAGGAG |
| *PERP7-like* | | TCCTGACTCCCACCATTGAA | GCGATACGAATCTTGCTCCT |
| *PER47* | | AAGTCCTCTCGTCTCCTCCT | GATCAAACCCTATTTGCCAGTG |
| *ATPb* | | AACAGGATTTGGCTCAGGATTG | AGACGCTACGGACTTAATTGGA |
| *TKL* | | AGAAAGTGAGGTGGAGAAAGTC | AAAGAGAGGGTTAGGGTGTGA |
